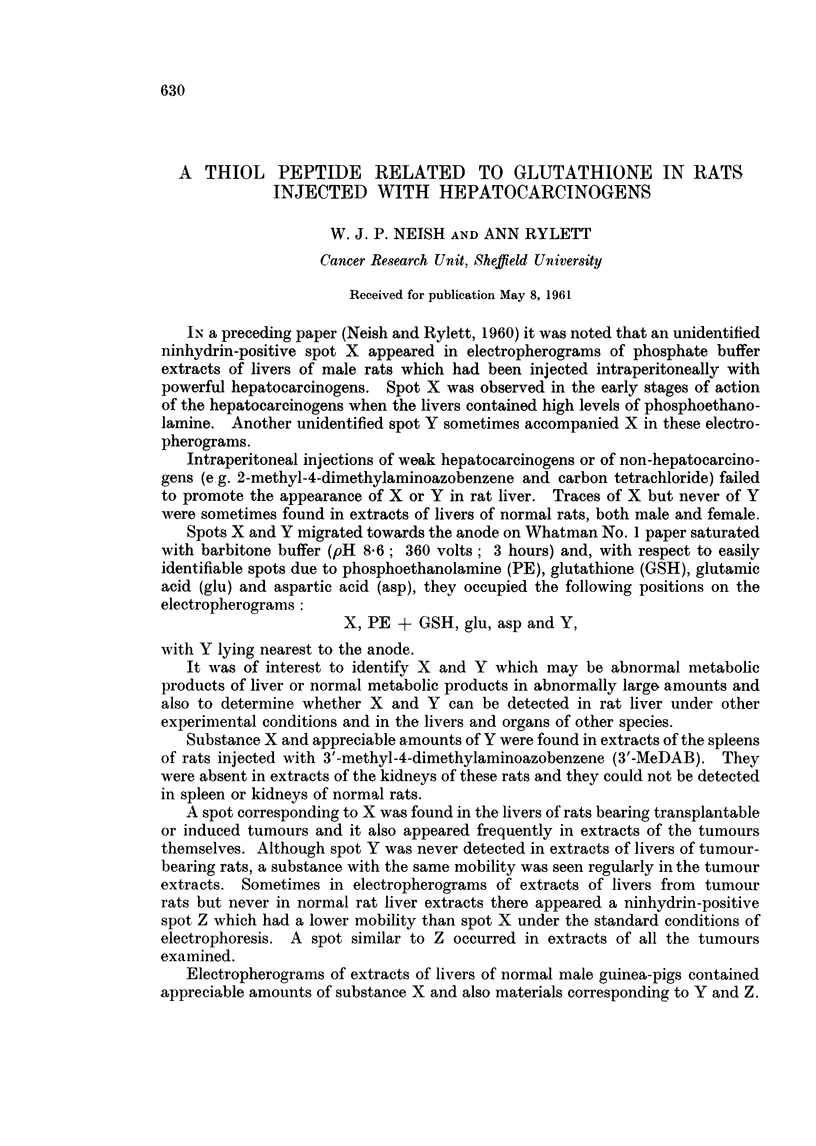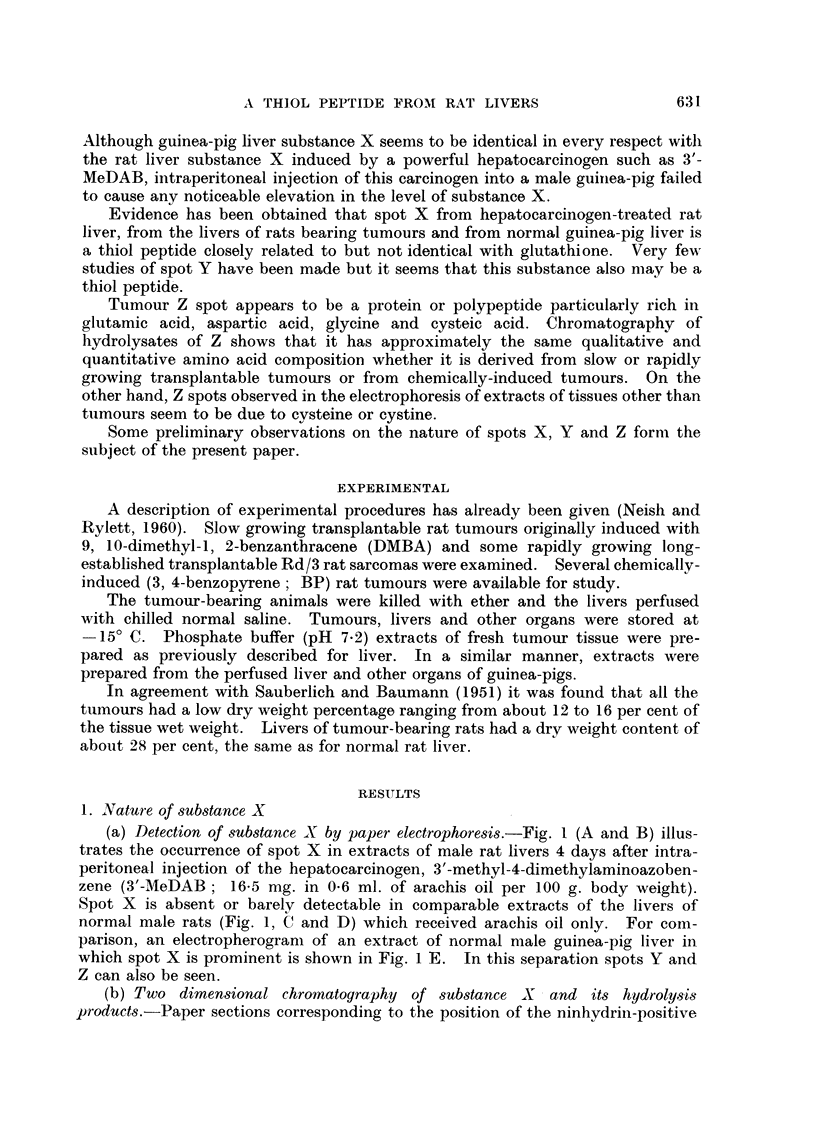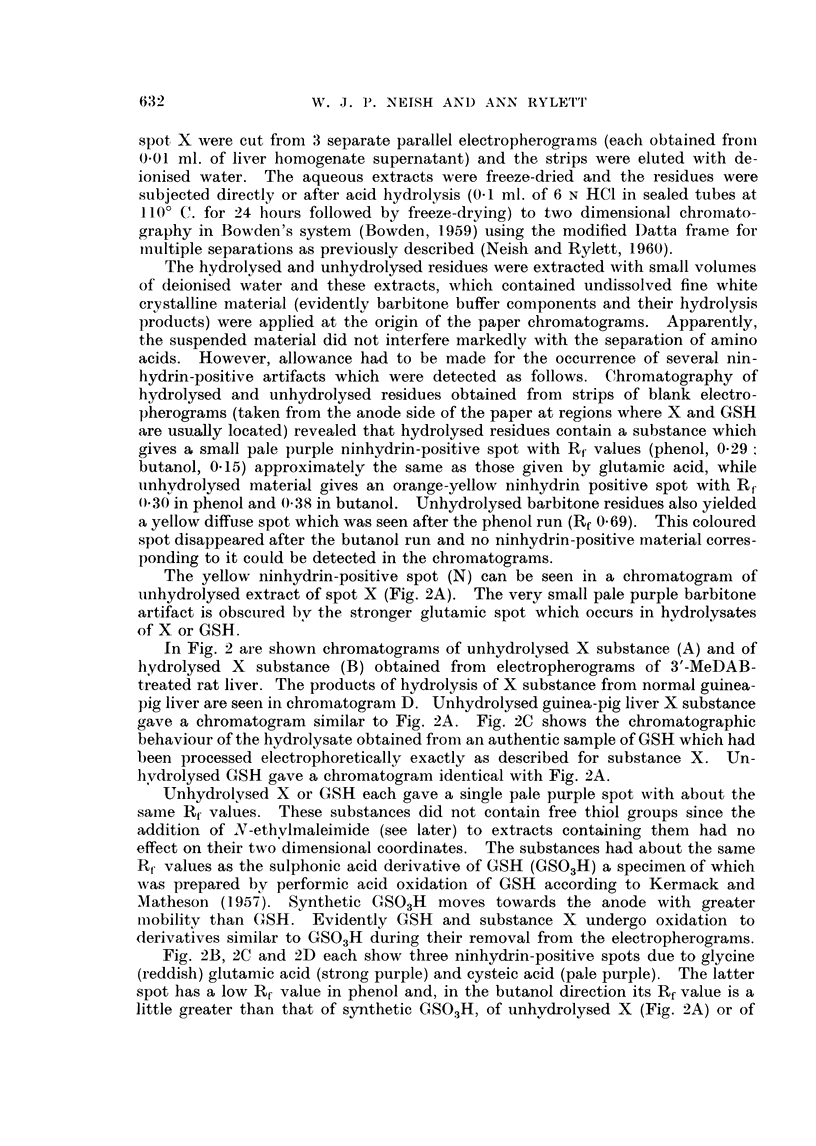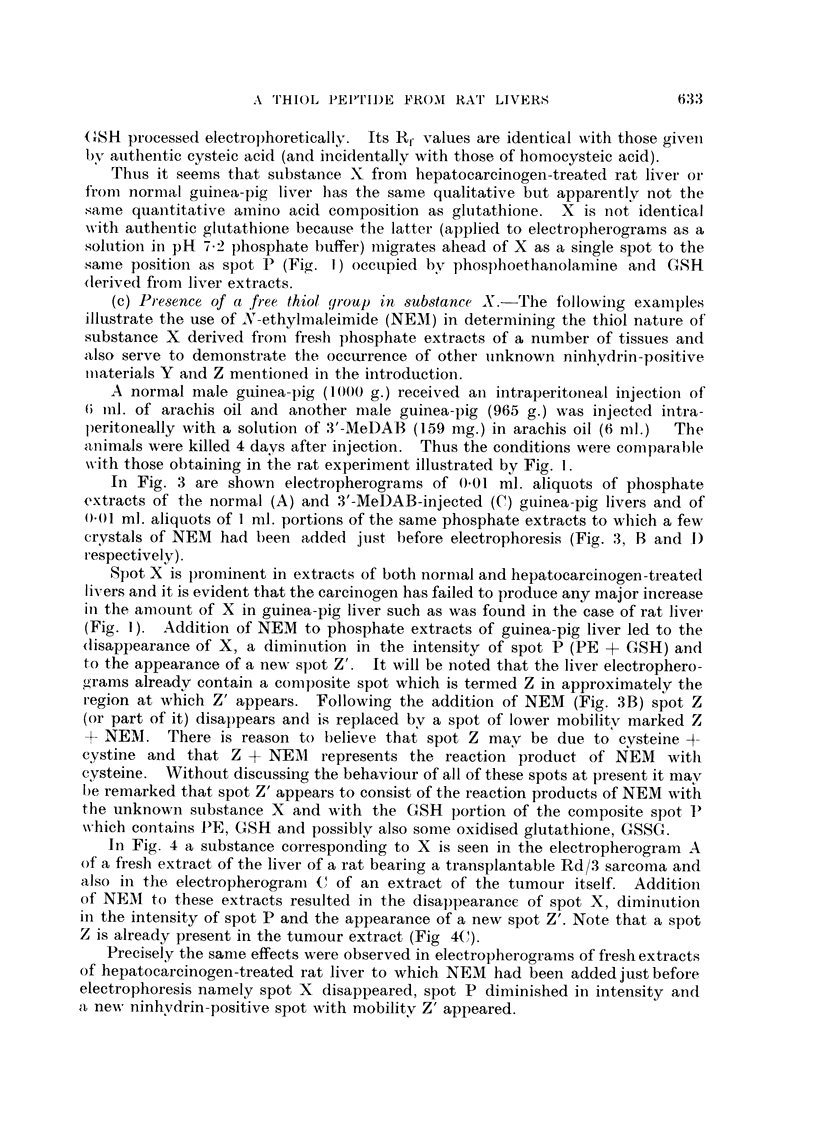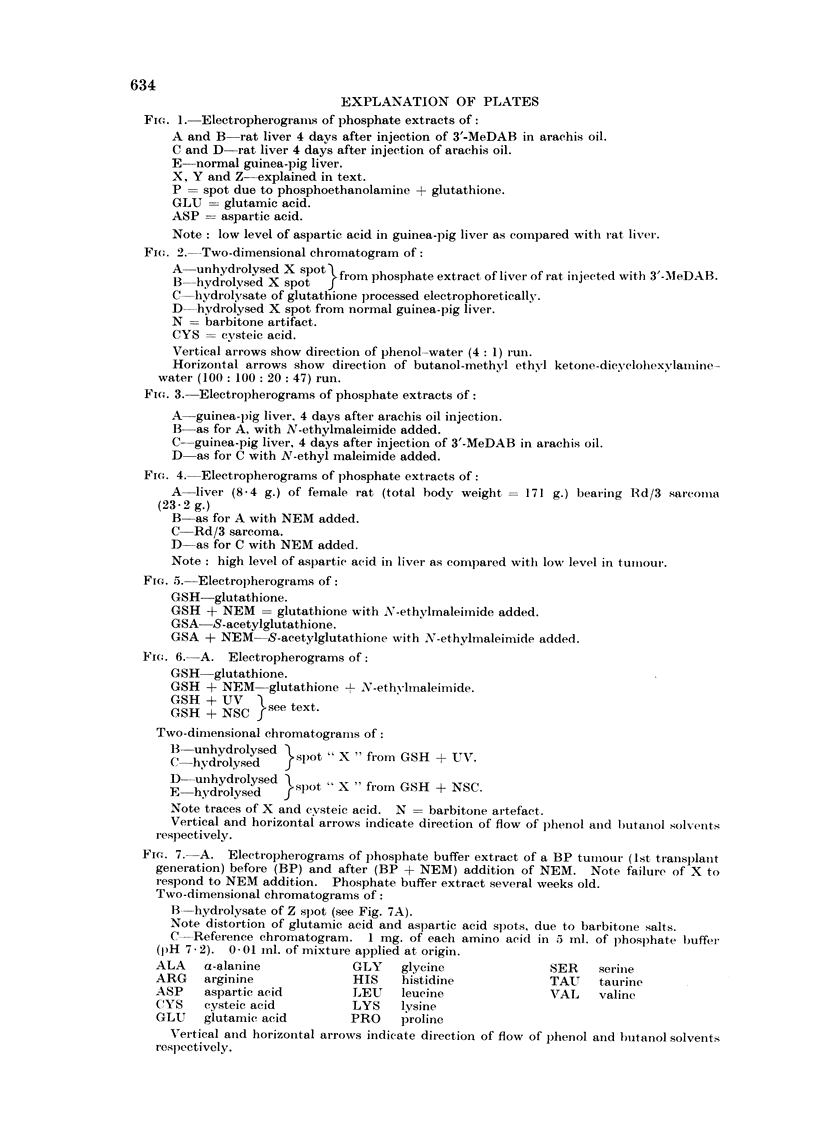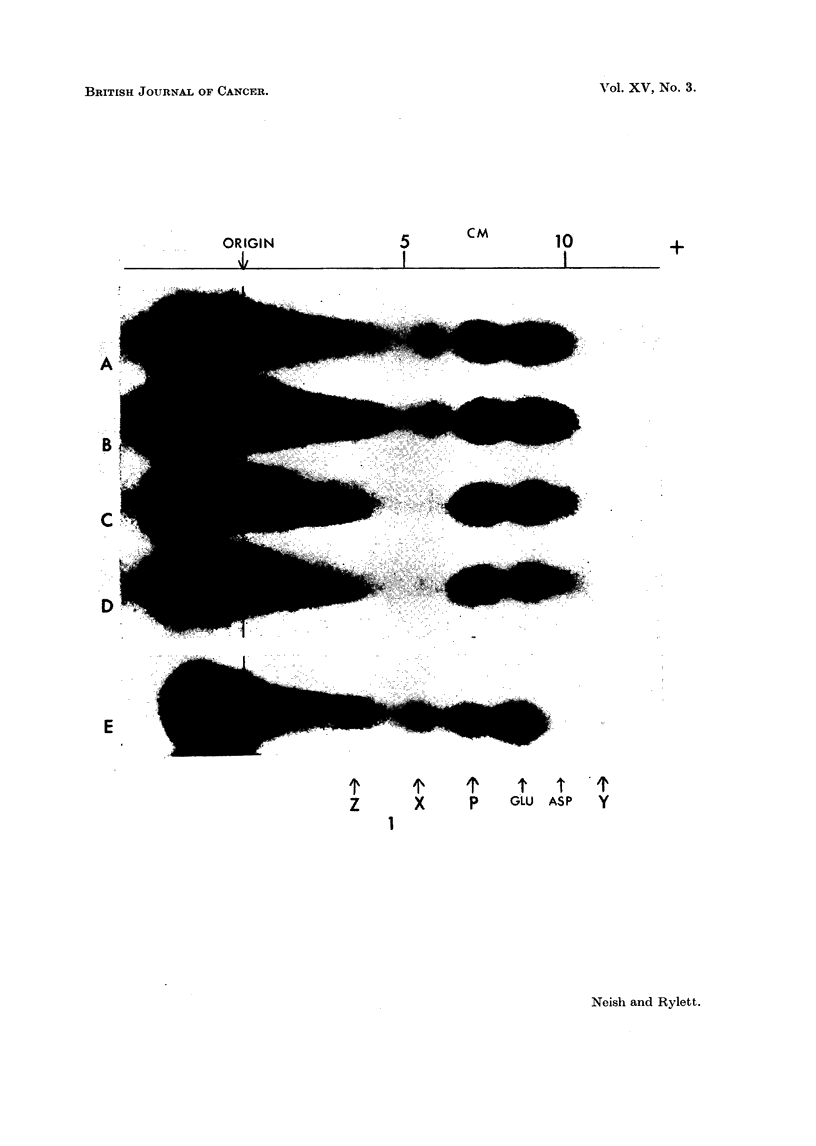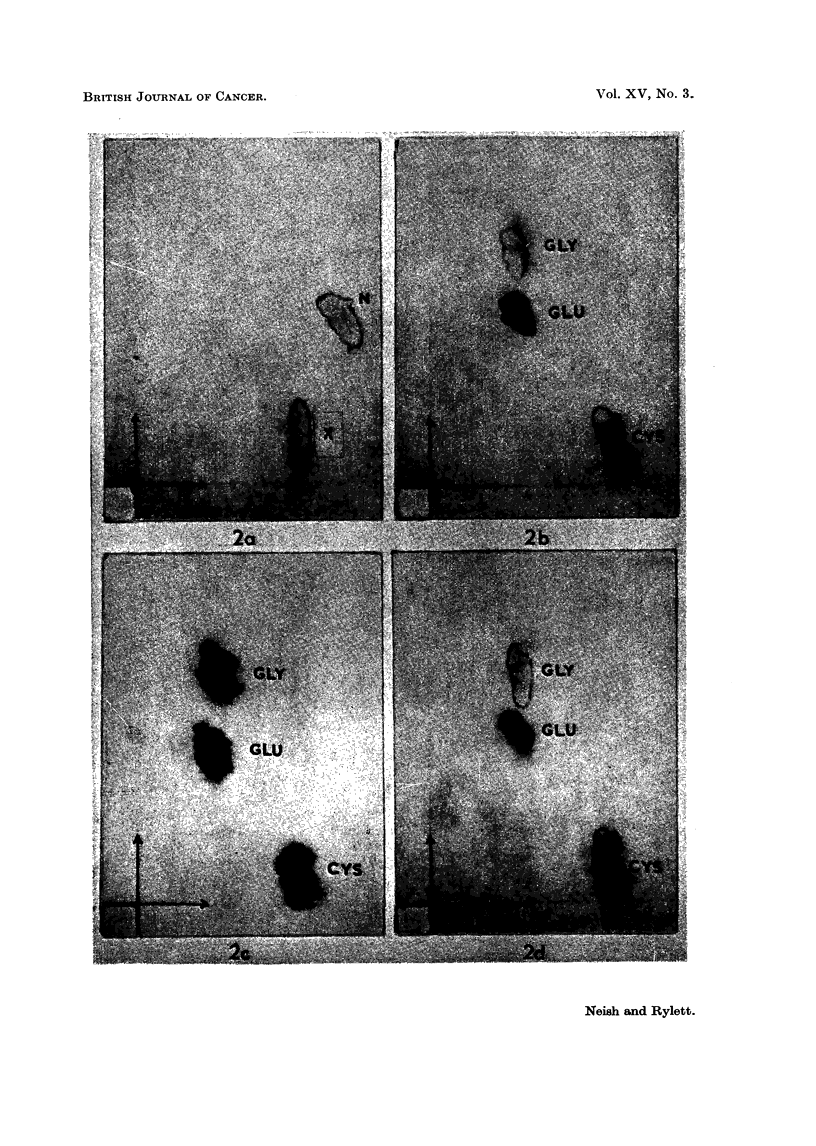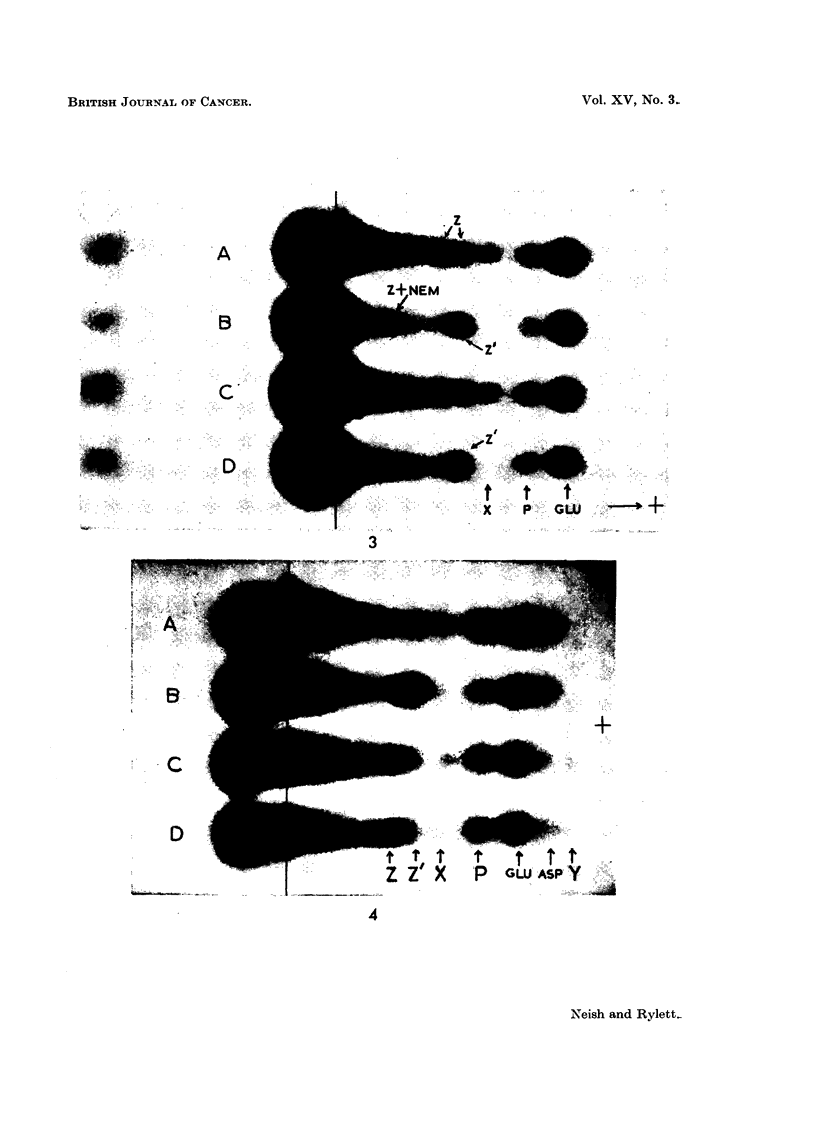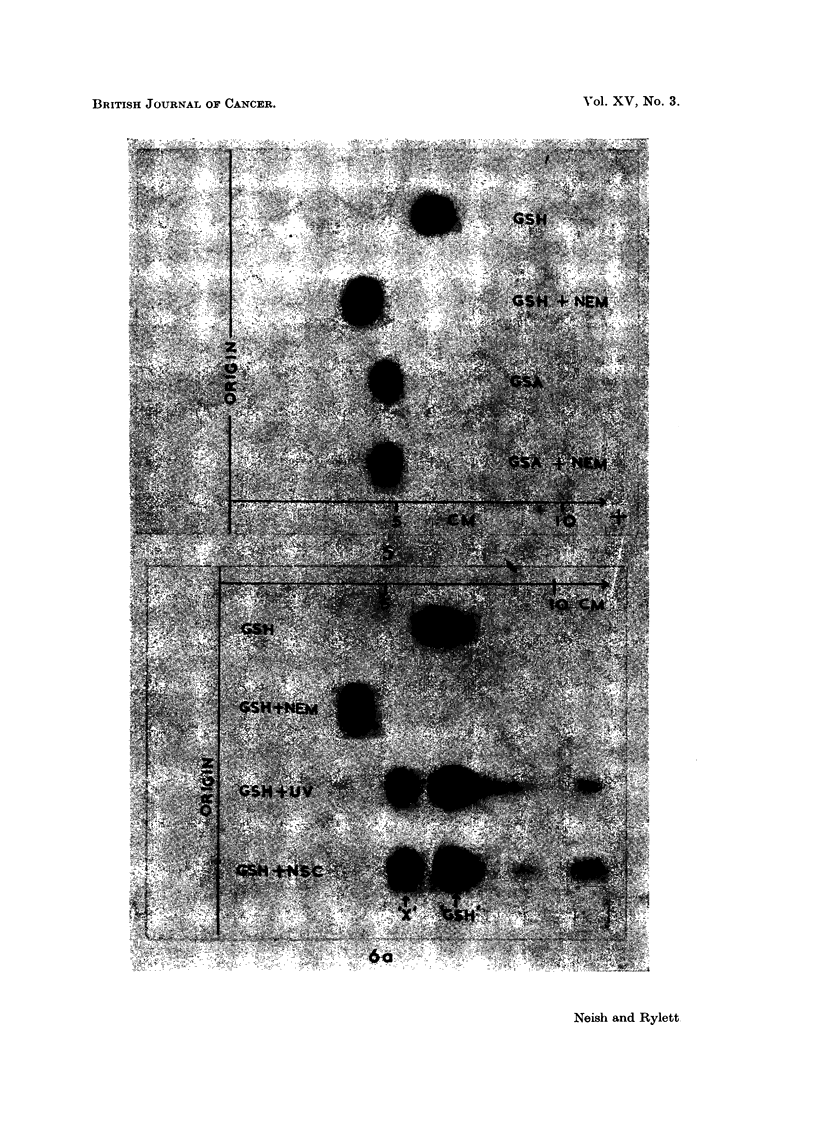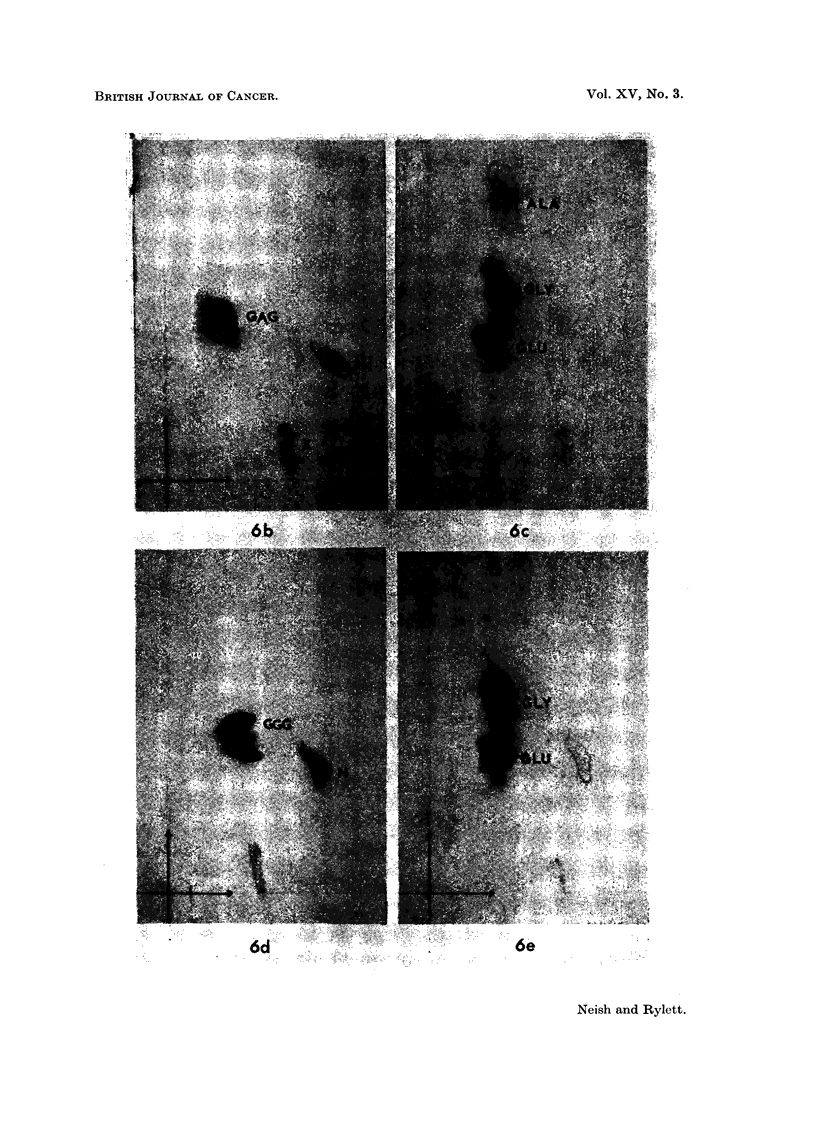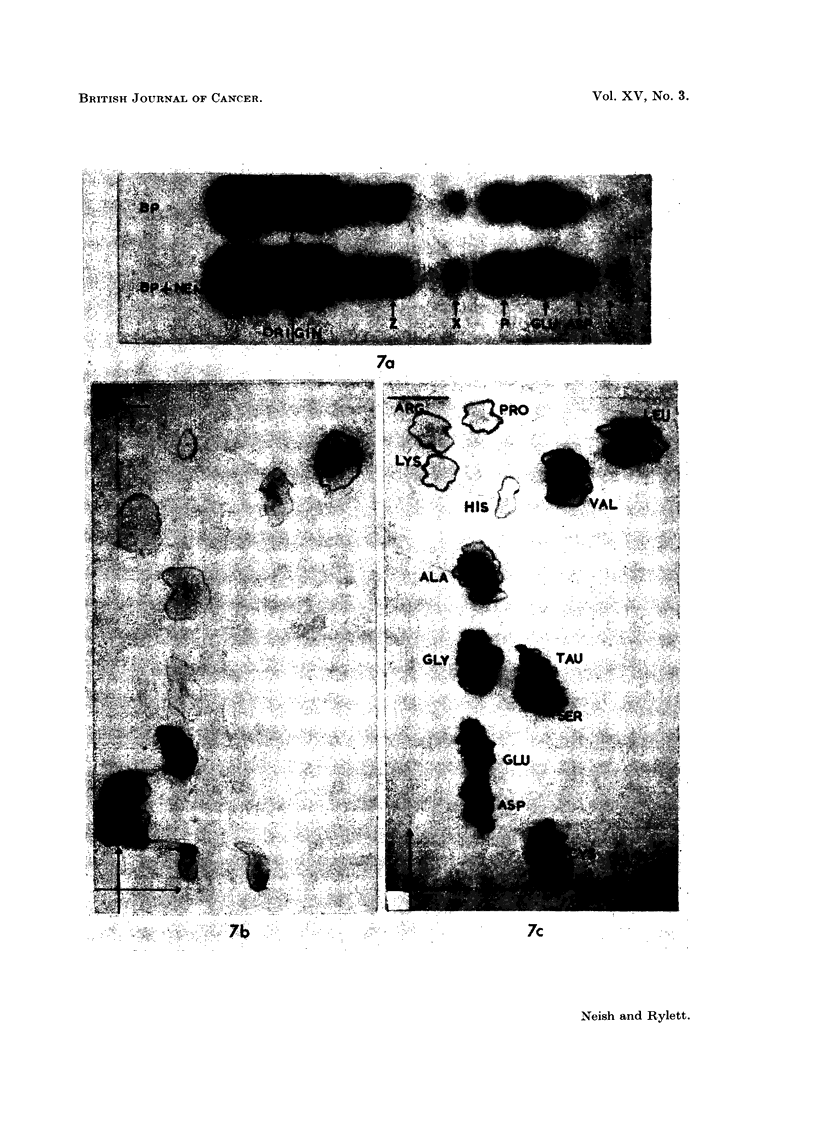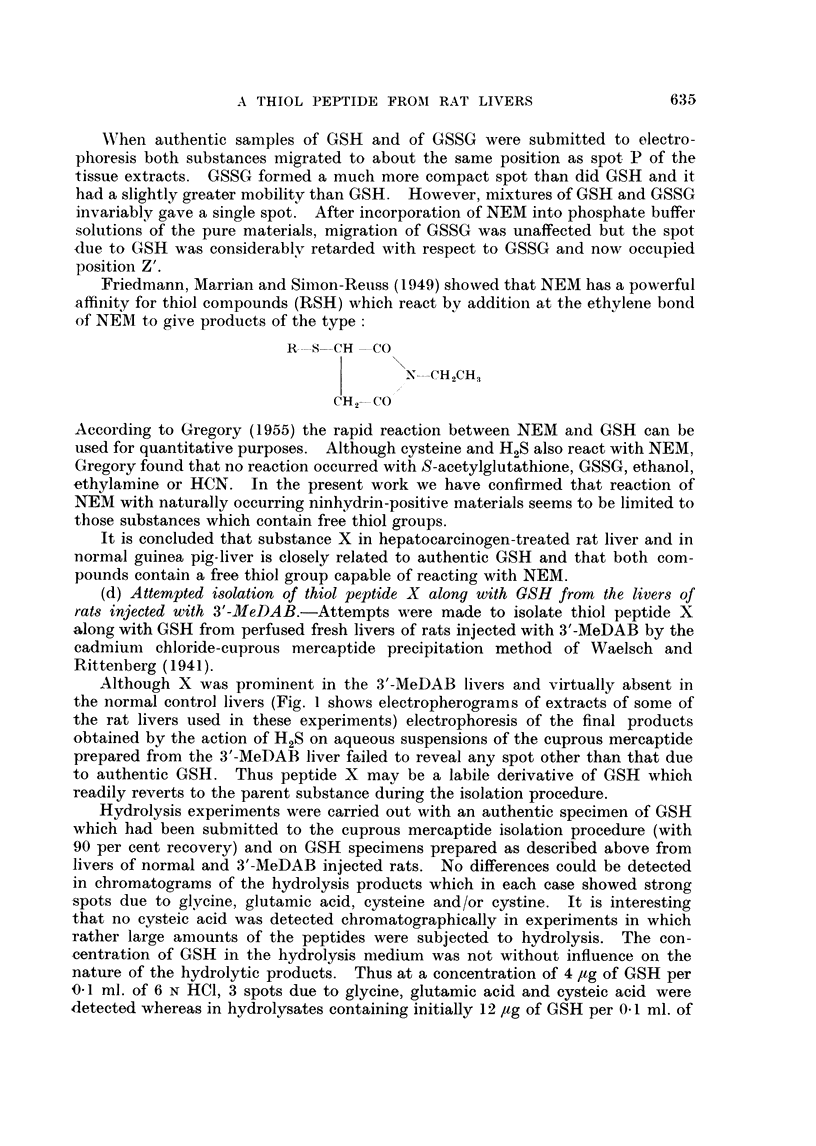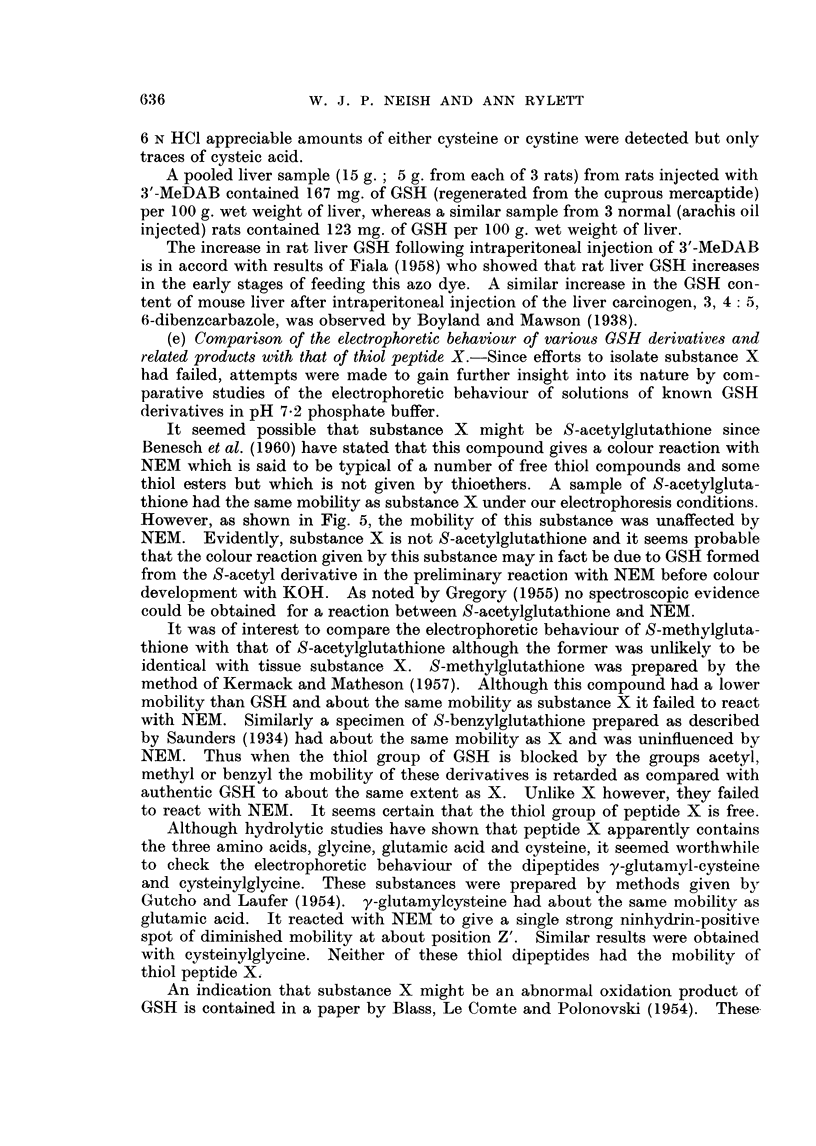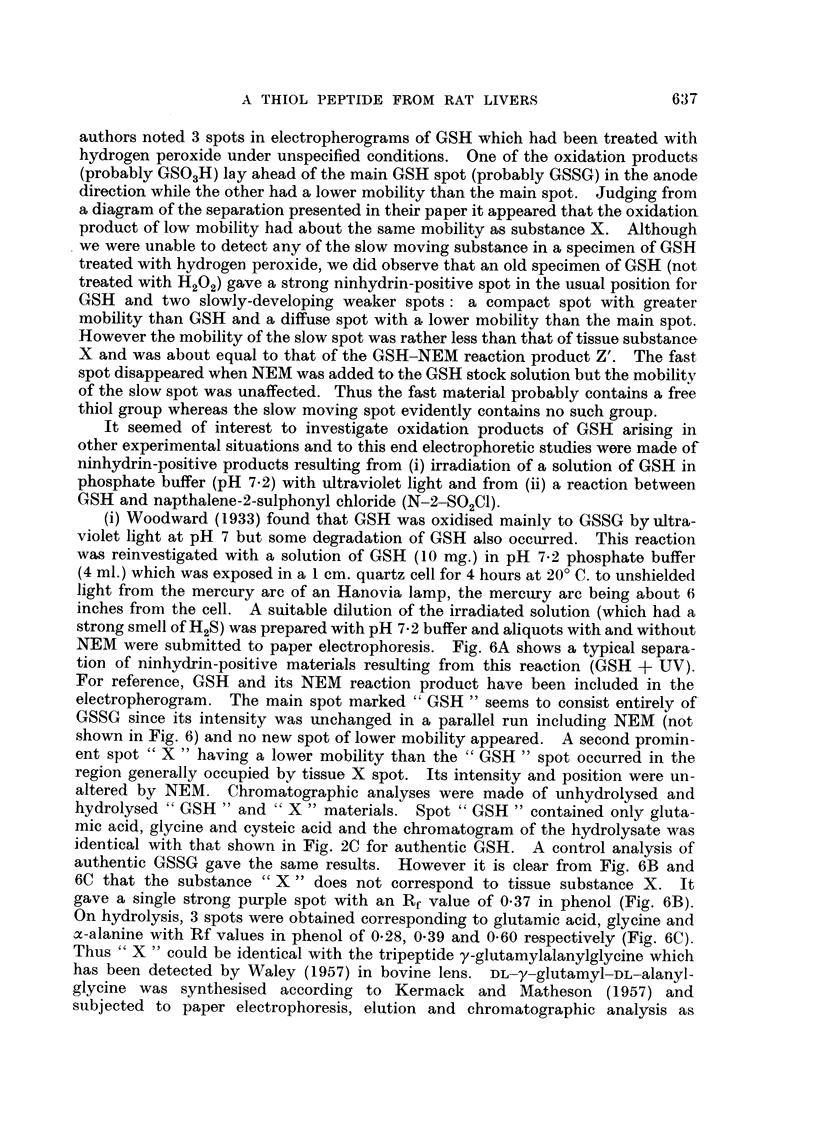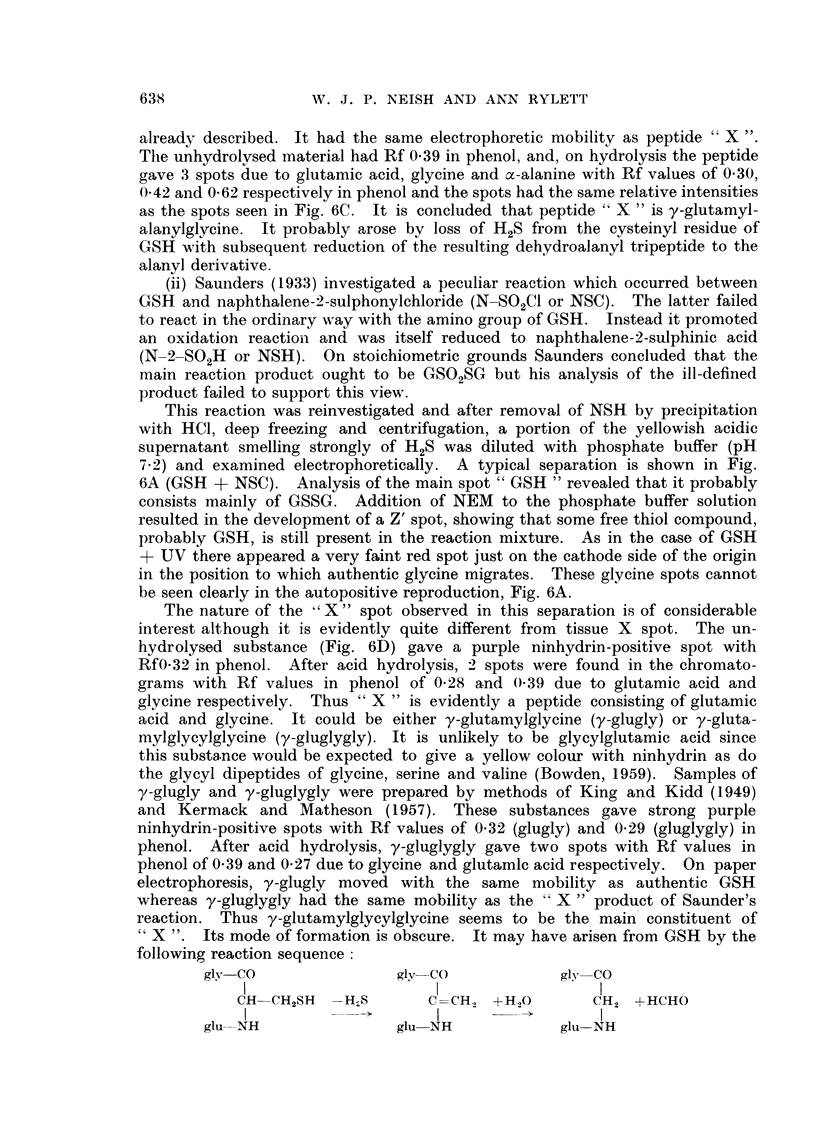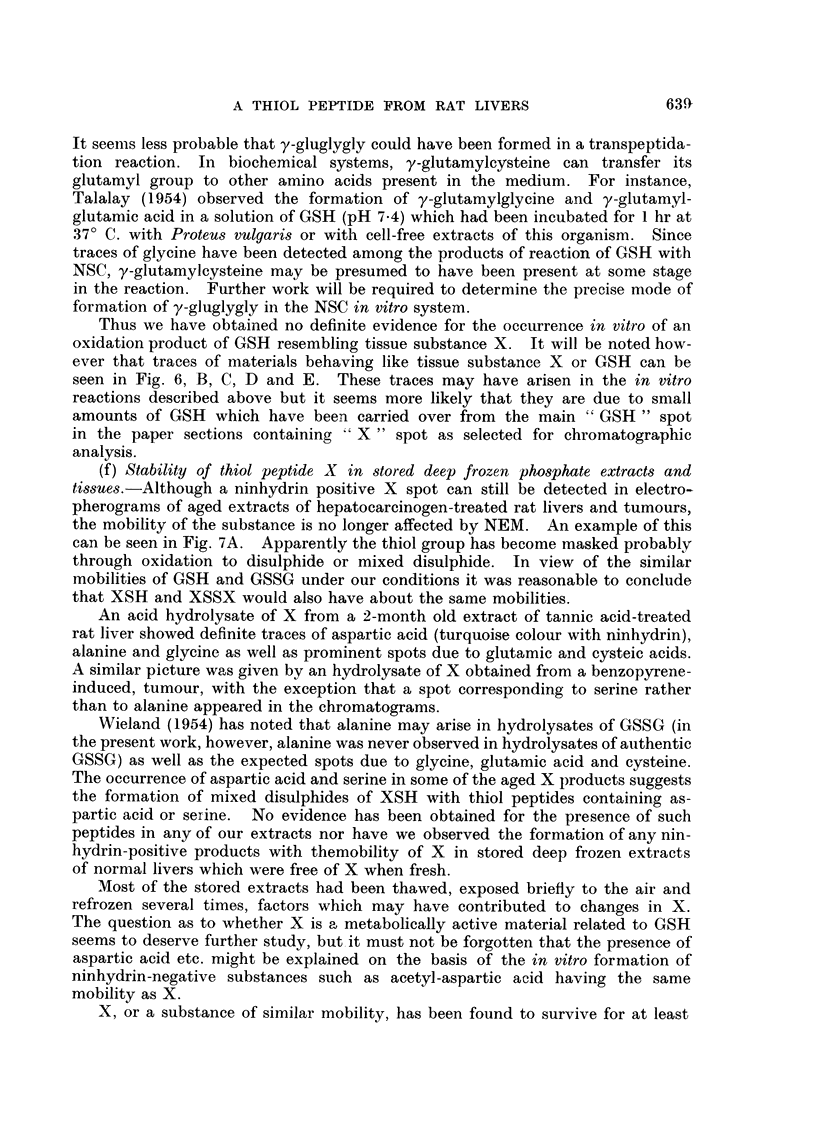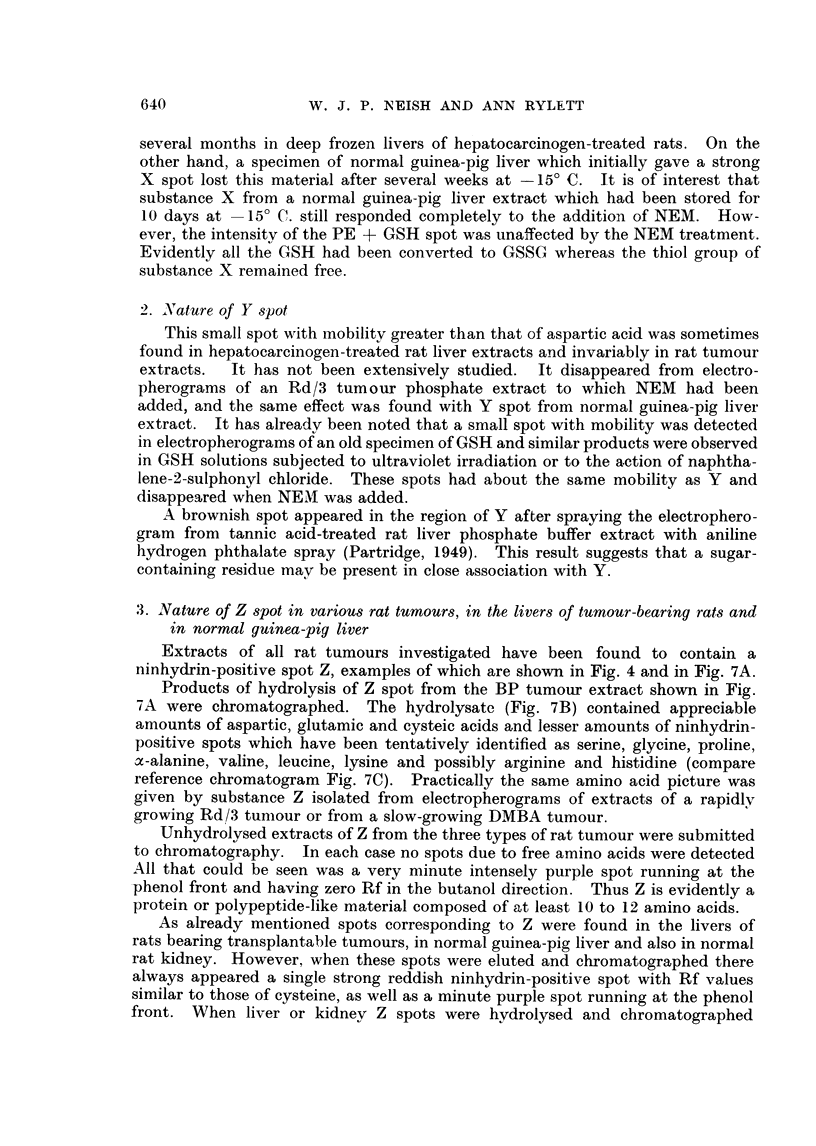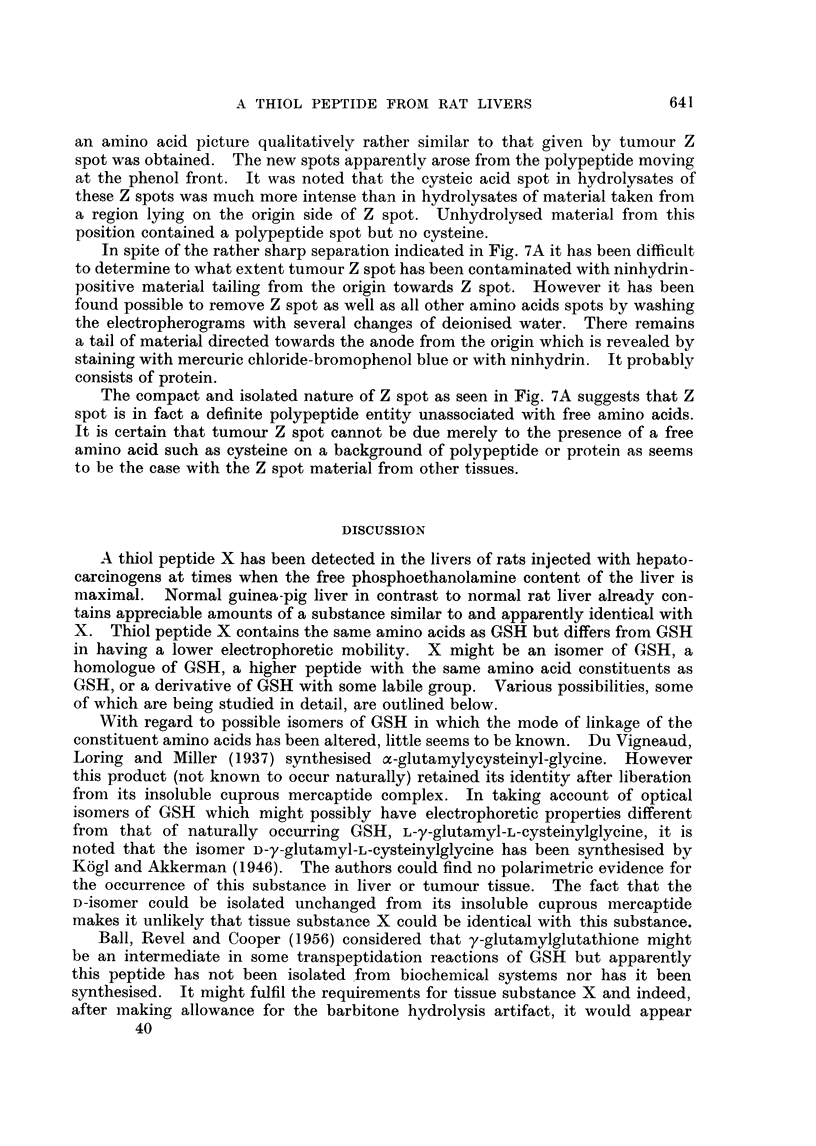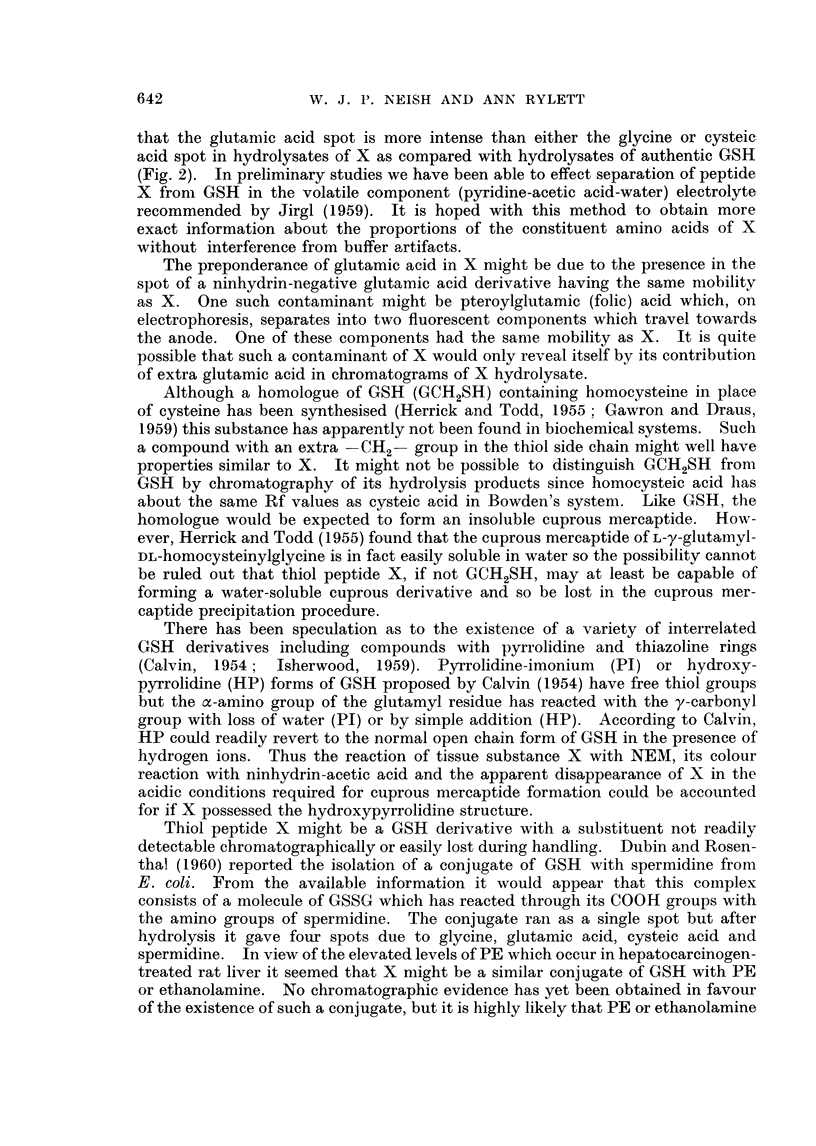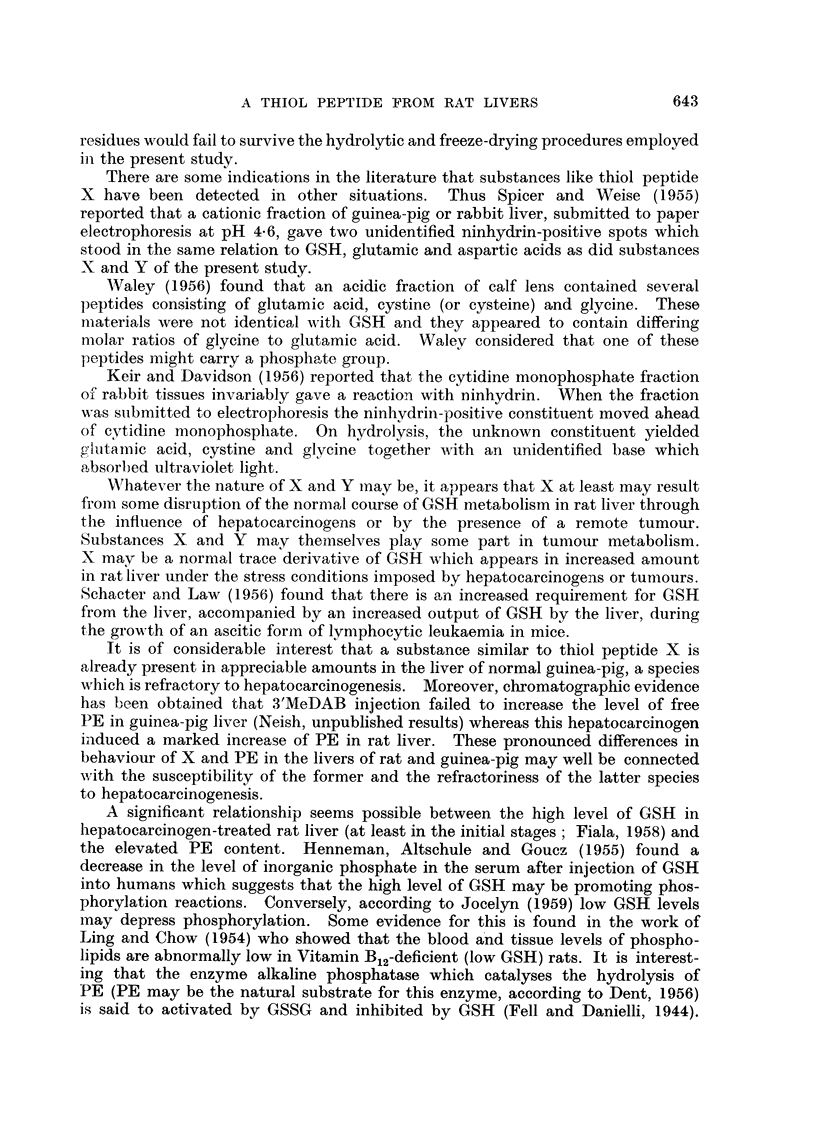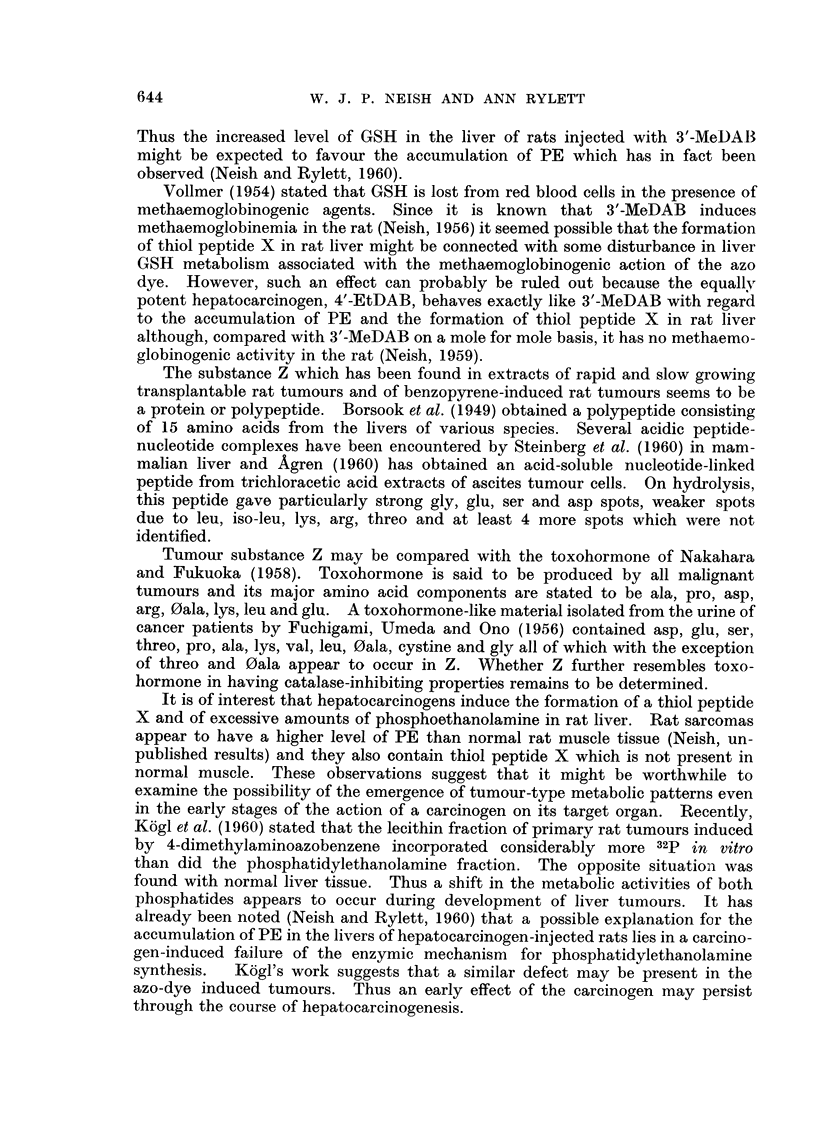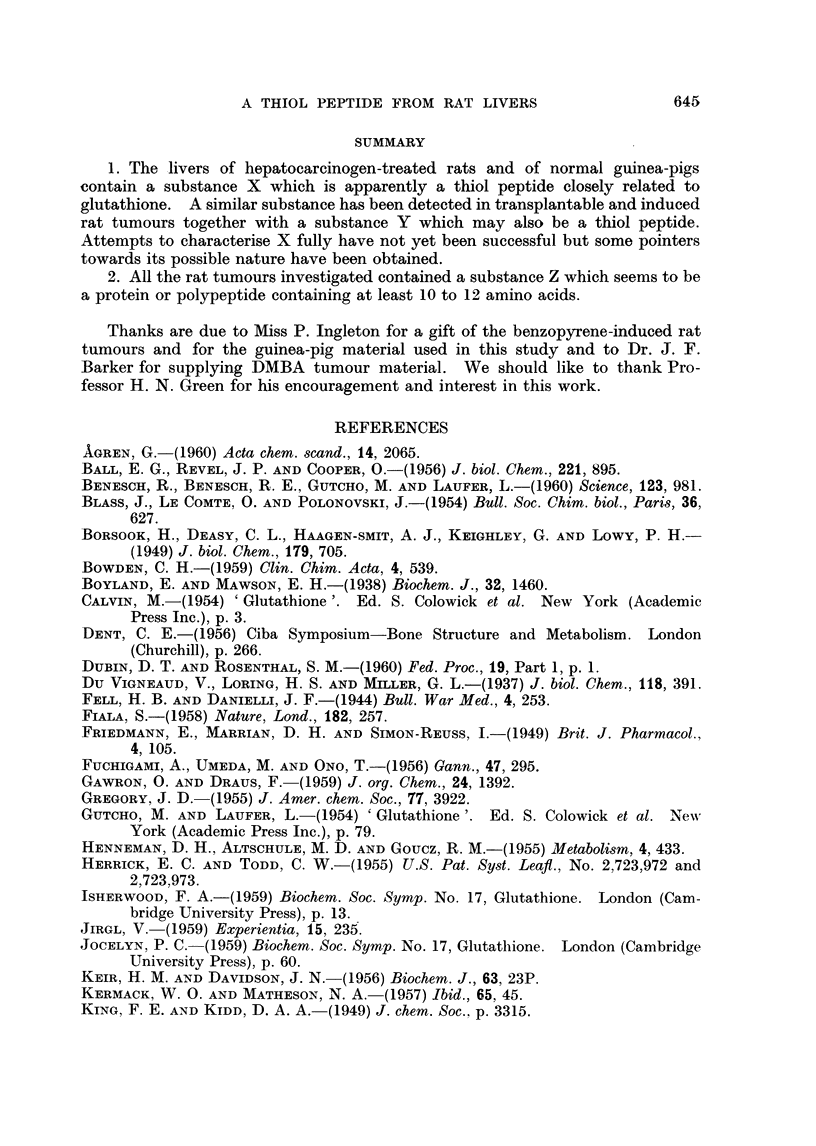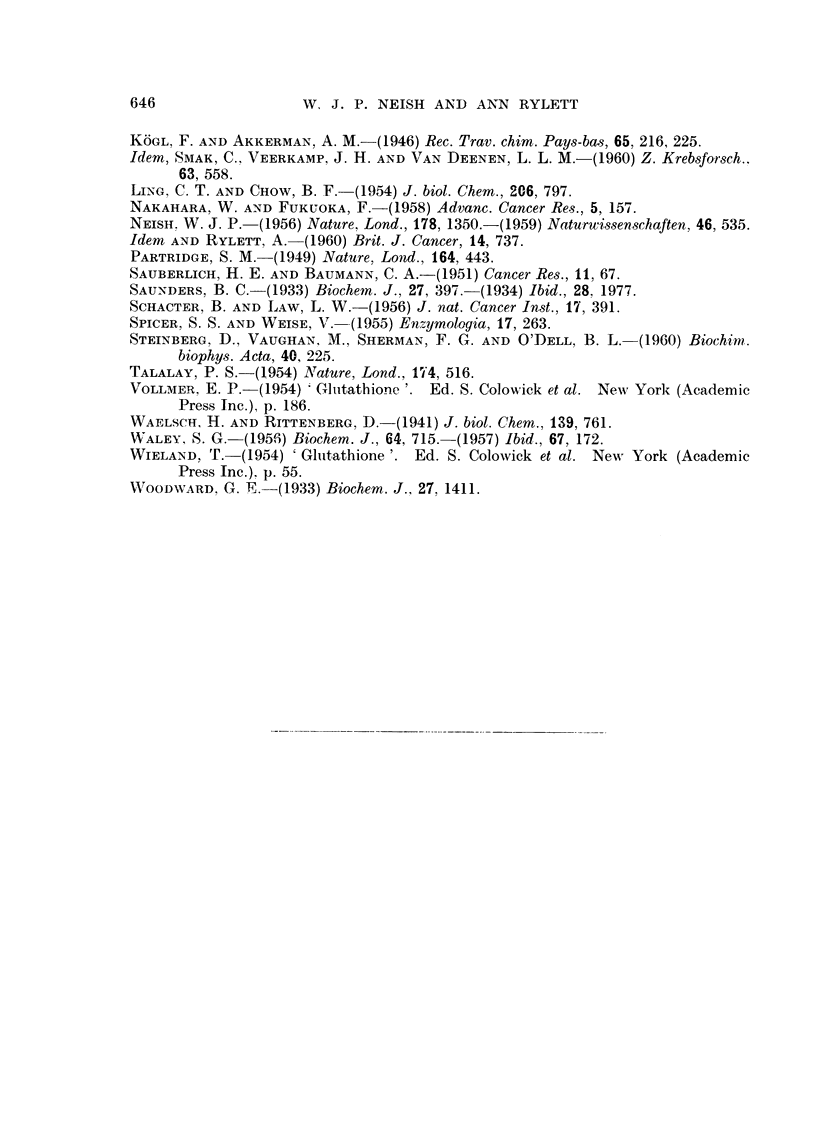# A Thiol Peptide related to Glutathione in Rats Injected with Hepatocarcinogens

**DOI:** 10.1038/bjc.1961.73

**Published:** 1961-09

**Authors:** W. J. P. Neish, Ann Rylett

## Abstract

**Images:**


### A THIOL PEPTIDE RELATED TO GLUTATHIONE IN RATS INJECTED WITH HEPATOCARCINOGENS

W. J. P. NEISH AND ANN RYLETT

*Cancer Research Unit, Sheffield University*

Received for publication May 8, 1961

IN a preceding paper (Neish and Rylett, 1960) it was noted that an unidentified ninhydrin-positive spot X appeared in electropherograms of phosphate buffer extracts of livers of male rats which had been injected intraperitoneally with powerful hepatocarcinogens. Spot X was observed in the early stages of action of the hepatocarcinogens when the livers contained high levels of phosphoethanolamine. Another unidentified spot Y sometimes accompanied X in these electropherograms.

Intraperitoneal injections of weak hepatocarcinogens or of non-hepatocarcinogens (e.g. 2-methyl-4-dimethylaminoazobenzene and carbon tetrachloride) failed to promote the appearance of X or Y in rat liver. Traces of X but never of Y were sometimes found in extracts of livers of normal rats, both male and female.

Spots X and Y migrated towards the anode on Whatman No. 1 paper saturated with barbitone buffer ($p$H 8·6 ; 360 volts ; 3 hours) and, with respect to easily identifiable spots due to phosphoethanolamine (PE), glutathione (GSH), glutamic acid (glu) and aspartic acid (asp), they occupied the following positions on the electropherograms :

X, PE + GSH, glu, asp and Y, with Y lying nearest to the anode.

It was of interest to identify X and Y which may be abnormal metabolic products of liver or normal metabolic products in abnormally large amounts and also to determine whether X and Y can be detected in rat liver under other experimental conditions and in the livers and organs of other species.

Substance X and appreciable amounts of Y were found in extracts of the spleens of rats injected with 3′-methyl-4-dimethylaminoazobenzene (3′-MeDAB). They were absent in extracts of the kidneys of these rats and they could not be detected in spleen or kidneys of normal rats.

A spot corresponding to X was found in the livers of rats bearing transplantable or induced tumours and it also appeared frequently in extracts of the tumours themselves. Although spot Y was never detected in extracts of livers of tumour-bearing rats, a substance with the same mobility was seen regularly in the tumour extracts. Sometimes in electropherograms of extracts of livers from tumour rats but never in normal rat liver extracts there appeared a ninhydrin-positive spot Z which had a lower mobility than spot X under the standard conditions of electrophoresis. A spot similar to Z occurred in extracts of all the tumours examined.

Electropherograms of extracts of livers of normal male guinea-pigs contained appreciable amounts of substance X and also materials corresponding to Y and Z.

Although guinea-pig liver substance X seems to be identical in every respect with the rat liver substance X induced by a powerful hepatocarcinogen such as 3′-MeDAB, intraperitoneal injection of this carcinogen into a male guinea-pig failed to cause any noticeable elevation in the level of substance X.

Evidence has been obtained that spot X from hepatocarcinogen-treated rat liver, from the livers of rats bearing tumours and from normal guinea-pig liver is a thiol peptide closely related to but not identical with glutathione. Very few studies of spot Y have been made but it seems that this substance also may be a thiol peptide.

Tumour Z spot appears to be a protein or polypeptide particularly rich in glutamic acid, aspartic acid, glycine and cysteic acid. Chromatography of hydrolysates of Z shows that it has approximately the same qualitative and quantitative amino acid composition whether it is derived from slow or rapidly growing transplantable tumours or from chemically-induced tumours. On the other hand, Z spots observed in the electrophoresis of extracts of tissues other than tumours seem to be due to cysteine or cystine.

Some preliminary observations on the nature of spots X, Y and Z form the subject of the present paper.

### EXPERIMENTAL

A description of experimental procedures has already been given (Neish and Rylett, 1960). Slow growing transplantable rat tumours originally induced with 9, 10-dimethyl-1, 2-benzanthracene (DMBA) and some rapidly growing long-established transplantable Rd/3 rat sarcomas were examined. Several chemically-induced (3, 4-benzopyrene ; BP) rat tumours were available for study.

The tumour-bearing animals were killed with ether and the livers perfused with chilled normal saline. Tumours, livers and other organs were stored at $-15°$ C. Phosphate buffer (pH 7·2) extracts of fresh tumour tissue were prepared as previously described for liver. In a similar manner, extracts were prepared from the perfused liver and other organs of guinea-pigs.

In agreement with Sauberlich and Baumann (1951) it was found that all the tumours had a low dry weight percentage ranging from about 12 to 16 per cent of the tissue wet weight. Livers of tumour-bearing rats had a dry weight content of about 28 per cent, the same as for normal rat liver.

### RESULTS

### 1. *Nature of substance X*

(a) *Detection of substance X by paper electrophoresis.*—Fig. 1 (A and B) illustrates the occurrence of spot X in extracts of male rat livers 4 days after intraperitoneal injection of the hepatocarcinogen, 3′-methyl-4-dimethylaminoazobenzene (3′-MeDAB ; 16·5 mg. in 0·6 ml. of arachis oil per 100 g. body weight). Spot X is absent or barely detectable in comparable extracts of the livers of normal male rats (Fig. 1, C and D) which received arachis oil only. For comparison, an electropherogram of an extract of normal male guinea-pig liver in which spot X is prominent is shown in Fig. 1 E. In this separation spots Y and Z can also be seen.

(b) *Two dimensional chromatography of substance X and its hydrolysis products.*—Paper sections corresponding to the position of the ninhydrin-positive spot X were cut from 3 separate parallel electropherograms (each obtained from 0·01 ml. of liver homogenate supernatant) and the strips were eluted with deionised water. The aqueous extracts were freeze-dried and the residues were subjected directly or after acid hydrolysis (0·1 ml. of 6 N HCl in sealed tubes at 110° C. for 24 hours followed by freeze-drying) to two dimensional chromatography in Bowden's system (Bowden, 1959) using the modified Datta frame for multiple separations as previously described (Neish and Rylett, 1960).

The hydrolysed and unhydrolysed residues were extracted with small volumes of deionised water and these extracts, which contained undissolved fine white crystalline material (evidently barbitone buffer components and their hydrolysis products) were applied at the origin of the paper chromatograms. Apparently, the suspended material did not interfere markedly with the separation of amino acids. However, allowance had to be made for the occurrence of several ninhydrin-positive artifacts which were detected as follows. Chromatography of hydrolysed and unhydrolysed residues obtained from strips of blank electropherograms (taken from the anode side of the paper at regions where X and GSH are usually located) revealed that hydrolysed residues contain a substance which gives a small pale purple ninhydrin-positive spot with $R_f$ values (phenol, 0·29 ; butanol, 0·15) approximately the same as those given by glutamic acid, while unhydrolysed material gives an orange-yellow ninhydrin positive spot with $R_f$ 0·30 in phenol and 0·38 in butanol. Unhydrolysed barbitone residues also yielded a yellow diffuse spot which was seen after the phenol run ($R_f$ 0·69). This coloured spot disappeared after the butanol run and no ninhydrin-positive material corresponding to it could be detected in the chromatograms.

The yellow ninhydrin-positive spot (N) can be seen in a chromatogram of unhydrolysed extract of spot X (Fig. 2A). The very small pale purple barbitone artifact is obscured by the stronger glutamic spot which occurs in hydrolysates of X or GSH.

In Fig. 2 are shown chromatograms of unhydrolysed X substance (A) and of hydrolysed X substance (B) obtained from electropherograms of 3′-MeDAB-treated rat liver. The products of hydrolysis of X substance from normal guinea-pig liver are seen in chromatogram D. Unhydrolysed guinea-pig liver X substance gave a chromatogram similar to Fig. 2A. Fig. 2C shows the chromatographic behaviour of the hydrolysate obtained from an authentic sample of GSH which had been processed electrophoretically exactly as described for substance X. Unhydrolysed GSH gave a chromatogram identical with Fig. 2A.

Unhydrolysed X or GSH each gave a single pale purple spot with about the same $R_f$ values. These substances did not contain free thiol groups since the addition of *N*-ethylmaleimide (see later) to extracts containing them had no effect on their two dimensional coordinates. The substances had about the same $R_f$ values as the sulphonic acid derivative of GSH ($GSO_3H$) a specimen of which was prepared by performic acid oxidation of GSH according to Kermack and Matheson (1957). Synthetic $GSO_3H$ moves towards the anode with greater mobility than GSH. Evidently GSH and substance X undergo oxidation to derivatives similar to $GSO_3H$ during their removal from the electropherograms.

Fig. 2B, 2C and 2D each show three ninhydrin-positive spots due to glycine (reddish) glutamic acid (strong purple) and cysteic acid (pale purple). The latter spot has a low $R_f$ value in phenol and, in the butanol direction its $R_f$ value is a little greater than that of synthetic $GSO_3H$, of unhydrolysed X (Fig. 2A) or of

GSH processed electrophoretically. Its $R_f$ values are identical with those given by authentic cysteic acid (and incidentally with those of homocysteic acid).

Thus it seems that substance X from hepatocarcinogen-treated rat liver or from normal guinea-pig liver has the same qualitative but apparently not the same quantitative amino acid composition as glutathione. X is not identical with authentic glutathione because the latter (applied to electropherograms as a solution in pH 7·2 phosphate buffer) migrates ahead of X as a single spot to the same position as spot P (Fig. 1) occupied by phosphoethanolamine and GSH derived from liver extracts.

(c) *Presence of a free thiol group in substance X.*—The following examples illustrate the use of *N*-ethylmaleimide (NEM) in determining the thiol nature of substance X derived from fresh phosphate extracts of a number of tissues and also serve to demonstrate the occurrence of other unknown ninhydrin-positive materials Y and Z mentioned in the introduction.

A normal male guinea-pig (1000 g.) received an intraperitoneal injection of 6 ml. of arachis oil and another male guinea-pig (965 g.) was injected intraperitoneally with a solution of 3′-MeDAB (159 mg.) in arachis oil (6 ml.) The animals were killed 4 days after injection. Thus the conditions were comparable with those obtaining in the rat experiment illustrated by Fig. 1.

In Fig. 3 are shown electropherograms of 0·01 ml. aliquots of phosphate extracts of the normal (A) and 3′-MeDAB-injected (C) guinea-pig livers and of 0·01 ml. aliquots of 1 ml. portions of the same phosphate extracts to which a few crystals of NEM had been added just before electrophoresis (Fig. 3, B and D respectively).

Spot X is prominent in extracts of both normal and hepatocarcinogen-treated livers and it is evident that the carcinogen has failed to produce any major increase in the amount of X in guinea-pig liver such as was found in the case of rat liver (Fig. 1). Addition of NEM to phosphate extracts of guinea-pig liver led to the disappearance of X, a diminution in the intensity of spot P (PE + GSH) and to the appearance of a new spot Z′. It will be noted that the liver electropherograms already contain a composite spot which is termed Z in approximately the region at which Z′ appears. Following the addition of NEM (Fig. 3B) spot Z (or part of it) disappears and is replaced by a spot of lower mobility marked Z + NEM. There is reason to believe that spot Z may be due to cysteine + cystine and that Z + NEM represents the reaction product of NEM with cysteine. Without discussing the behaviour of all of these spots at present it may be remarked that spot Z′ appears to consist of the reaction products of NEM with the unknown substance X and with the GSH portion of the composite spot P which contains PE, GSH and possibly also some oxidised glutathione, GSSG.

In Fig. 4 a substance corresponding to X is seen in the electropherogram A of a fresh extract of the liver of a rat bearing a transplantable Rd/3 sarcoma and also in the electropherogram C of an extract of the tumour itself. Addition of NEM to these extracts resulted in the disappearance of spot X, diminution in the intensity of spot P and the appearance of a new spot Z′. Note that a spot Z is already present in the tumour extract (Fig 4C).

Precisely the same effects were observed in electropherograms of fresh extracts of hepatocarcinogen-treated rat liver to which NEM had been added just before electrophoresis namely spot X disappeared, spot P diminished in intensity and a new ninhydrin-positive spot with mobility Z′ appeared.

### EXPLANATION OF PLATES

FIG. 1.—Electropherograms of phosphate extracts of :

A and B—rat liver 4 days after injection of 3′-MeDAB in arachis oil.
C and D—rat liver 4 days after injection of arachis oil.
E—normal guinea-pig liver.
X, Y and Z—explained in text.
P = spot due to phosphoethanolamine + glutathione.
GLU = glutamic acid.
ASP = aspartic acid.

Note : low level of aspartic acid in guinea-pig liver as compared with rat liver.

FIG. 2.—Two-dimensional chromatogram of :

A—unhydrolysed X spot } from phosphate extract of liver of rat injected with 3′-MeDAB.
B—hydrolysed X spot } from phosphate extract of liver of rat injected with 3′-MeDAB.
C—hydrolysate of glutathione processed electrophoretically.
D—hydrolysed X spot from normal guinea-pig liver.
N = barbitone artifact.
CYS = cysteic acid.

Vertical arrows show direction of phenol-water (4 : 1) run.
Horizontal arrows show direction of butanol-methyl ethyl ketone-dicyclohexylamine-water (100 : 100 : 20 : 47) run.

FIG. 3.—Electropherograms of phosphate extracts of :

A—guinea-pig liver, 4 days after arachis oil injection.
B—as for A, with *N*-ethylmaleimide added.
C—guinea-pig liver, 4 days after injection of 3′-MeDAB in arachis oil.
D—as for C with *N*-ethyl maleimide added.

FIG. 4.—Electropherograms of phosphate extracts of :

A—liver (8·4 g.) of female rat (total body weight = 171 g.) bearing Rd/3 sarcoma (23·2 g.)
B—as for A with NEM added.
C—Rd/3 sarcoma.
D—as for C with NEM added.

Note : high level of aspartic acid in liver as compared with low level in tumour.

FIG. 5.—Electropherograms of :

GSH—glutathione.
GSH + NEM = glutathione with *N*-ethylmaleimide added.
GSA—*S*-acetylglutathione.
GSA + NEM—*S*-acetylglutathione with *N*-ethylmaleimide added.

FIG. 6.—A. Electropherograms of :

GSH—glutathione.

GSH + NEM—glutathione + *N*-ethylmaleimide.

GSH + UV } see text.

GSH + NSC } see text.

Two-dimensional chromatograms of :

B—unhydrolysed } spot " X " from GSH + UV.

C—hydrolysed } spot " X " from GSH + UV.

D—unhydrolysed } spot " X " from GSH + NSC.

E—hydrolysed } spot " X " from GSH + NSC.

Note traces of X and cysteic acid. N = barbitone artefact.
Vertical and horizontal arrows indicate direction of flow of phenol and butanol solvents respectively.

FIG. 7.—A. Electropherograms of phosphate buffer extract of a BP tumour (1st transplant generation) before (BP) and after (BP + NEM) addition of NEM. Note failure of X to respond to NEM addition. Phosphate buffer extract several weeks old.

Two-dimensional chromatograms of :

B—hydrolysate of Z spot (see Fig. 7A).
Note distortion of glutamic acid and aspartic acid spots, due to barbitone salts.
C—Reference chromatogram. 1 mg. of each amino acid in 5 ml. of phosphate buffer (pH 7·2). 0·01 ml. of mixture applied at origin.

| | | | | | |
|---|---|---|---|---|---|
| ALA | α-alanine | GLY | glycine | SER | serine |
| ARG | arginine | HIS | histidine | TAU | taurine |
| ASP | aspartic acid | LEU | leucine | VAL | valine |
| CYS | cysteic acid | LYS | lysine | | |
| GLU | glutamic acid | PRO | proline | | |

Vertical and horizontal arrows indicate direction of flow of phenol and butanol solvents respectively.

1

Neish and Rylett.

3

4

5

6a

Neish and Rylett

GAG
N
X
6b
ALA
GLY
GLU
6c
GGG
N
6d
GLY
GLU
6e

BP
BP+NEM
ORIGIN
Z
X
P
GLU
ASP
Y
+
ARG
PRO
LEU
LYS
HIS
VAL
ALA
GLY
TAU
SER
GLU
ASP
CYS

7a

7b

7c

When authentic samples of GSH and of GSSG were submitted to electrophoresis both substances migrated to about the same position as spot P of the tissue extracts. GSSG formed a much more compact spot than did GSH and it had a slightly greater mobility than GSH. However, mixtures of GSH and GSSG invariably gave a single spot. After incorporation of NEM into phosphate buffer solutions of the pure materials, migration of GSSG was unaffected but the spot due to GSH was considerably retarded with respect to GSSG and now occupied position Z′.

Friedmann, Marrian and Simon-Reuss (1949) showed that NEM has a powerful affinity for thiol compounds (RSH) which react by addition at the ethylene bond of NEM to give products of the type :

According to Gregory (1955) the rapid reaction between NEM and GSH can be used for quantitative purposes. Although cysteine and $H_2S$ also react with NEM, Gregory found that no reaction occurred with *S*-acetylglutathione, GSSG, ethanol, ethylamine or HCN. In the present work we have confirmed that reaction of NEM with naturally occurring ninhydrin-positive materials seems to be limited to those substances which contain free thiol groups.

It is concluded that substance X in hepatocarcinogen-treated rat liver and in normal guinea pig-liver is closely related to authentic GSH and that both compounds contain a free thiol group capable of reacting with NEM.

(d) *Attempted isolation of thiol peptide X along with GSH from the livers of rats injected with 3′-MeDAB.*—Attempts were made to isolate thiol peptide X along with GSH from perfused fresh livers of rats injected with 3′-MeDAB by the cadmium chloride-cuprous mercaptide precipitation method of Waelsch and Rittenberg (1941).

Although X was prominent in the 3′-MeDAB livers and virtually absent in the normal control livers (Fig. 1 shows electropherograms of extracts of some of the rat livers used in these experiments) electrophoresis of the final products obtained by the action of $H_2S$ on aqueous suspensions of the cuprous mercaptide prepared from the 3′-MeDAB liver failed to reveal any spot other than that due to authentic GSH. Thus peptide X may be a labile derivative of GSH which readily reverts to the parent substance during the isolation procedure.

Hydrolysis experiments were carried out with an authentic specimen of GSH which had been submitted to the cuprous mercaptide isolation procedure (with 90 per cent recovery) and on GSH specimens prepared as described above from livers of normal and 3′-MeDAB injected rats. No differences could be detected in chromatograms of the hydrolysis products which in each case showed strong spots due to glycine, glutamic acid, cysteine and/or cystine. It is interesting that no cysteic acid was detected chromatographically in experiments in which rather large amounts of the peptides were subjected to hydrolysis. The concentration of GSH in the hydrolysis medium was not without influence on the nature of the hydrolytic products. Thus at a concentration of 4 $\mu$g of GSH per 0·1 ml. of 6 N HCl, 3 spots due to glycine, glutamic acid and cysteic acid were detected whereas in hydrolysates containing initially 12 $\mu$g of GSH per 0·1 ml. of

6 N HCl appreciable amounts of either cysteine or cystine were detected but only traces of cysteic acid.

A pooled liver sample (15 g.; 5 g. from each of 3 rats) from rats injected with 3′-MeDAB contained 167 mg. of GSH (regenerated from the cuprous mercaptide) per 100 g. wet weight of liver, whereas a similar sample from 3 normal (arachis oil injected) rats contained 123 mg. of GSH per 100 g. wet weight of liver.

The increase in rat liver GSH following intraperitoneal injection of 3′-MeDAB is in accord with results of Fiala (1958) who showed that rat liver GSH increases in the early stages of feeding this azo dye. A similar increase in the GSH content of mouse liver after intraperitoneal injection of the liver carcinogen, 3, 4 : 5, 6-dibenzcarbazole, was observed by Boyland and Mawson (1938).

(e) *Comparison of the electrophoretic behaviour of various GSH derivatives and related products with that of thiol peptide X.*—Since efforts to isolate substance X had failed, attempts were made to gain further insight into its nature by comparative studies of the electrophoretic behaviour of solutions of known GSH derivatives in pH 7·2 phosphate buffer.

It seemed possible that substance X might be *S*-acetylglutathione since Benesch *et al.* (1960) have stated that this compound gives a colour reaction with NEM which is said to be typical of a number of free thiol compounds and some thiol esters but which is not given by thioethers. A sample of *S*-acetylglutathione had the same mobility as substance X under our electrophoresis conditions. However, as shown in Fig. 5, the mobility of this substance was unaffected by NEM. Evidently, substance X is not *S*-acetylglutathione and it seems probable that the colour reaction given by this substance may in fact be due to GSH formed from the *S*-acetyl derivative in the preliminary reaction with NEM before colour development with KOH. As noted by Gregory (1955) no spectroscopic evidence could be obtained for a reaction between *S*-acetylglutathione and NEM.

It was of interest to compare the electrophoretic behaviour of *S*-methylglutathione with that of *S*-acetylglutathione although the former was unlikely to be identical with tissue substance X. *S*-methylglutathione was prepared by the method of Kermack and Matheson (1957). Although this compound had a lower mobility than GSH and about the same mobility as substance X it failed to react with NEM. Similarly a specimen of *S*-benzylglutathione prepared as described by Saunders (1934) had about the same mobility as X and was uninfluenced by NEM. Thus when the thiol group of GSH is blocked by the groups acetyl, methyl or benzyl the mobility of these derivatives is retarded as compared with authentic GSH to about the same extent as X. Unlike X however, they failed to react with NEM. It seems certain that the thiol group of peptide X is free.

Although hydrolytic studies have shown that peptide X apparently contains the three amino acids, glycine, glutamic acid and cysteine, it seemed worthwhile to check the electrophoretic behaviour of the dipeptides $\gamma$-glutamyl-cysteine and cysteinylglycine. These substances were prepared by methods given by Gutcho and Laufer (1954). $\gamma$-glutamylcysteine had about the same mobility as glutamic acid. It reacted with NEM to give a single strong ninhydrin-positive spot of diminished mobility at about position Z′. Similar results were obtained with cysteinylglycine. Neither of these thiol dipeptides had the mobility of thiol peptide X.

An indication that substance X might be an abnormal oxidation product of GSH is contained in a paper by Blass, Le Comte and Polonovski (1954). These authors noted 3 spots in electropherograms of GSH which had been treated with hydrogen peroxide under unspecified conditions. One of the oxidation products (probably $GSO_3H$) lay ahead of the main GSH spot (probably GSSG) in the anode direction while the other had a lower mobility than the main spot. Judging from a diagram of the separation presented in their paper it appeared that the oxidation product of low mobility had about the same mobility as substance X. Although we were unable to detect any of the slow moving substance in a specimen of GSH treated with hydrogen peroxide, we did observe that an old specimen of GSH (not treated with $H_2O_2$) gave a strong ninhydrin-positive spot in the usual position for GSH and two slowly-developing weaker spots : a compact spot with greater mobility than GSH and a diffuse spot with a lower mobility than the main spot. However the mobility of the slow spot was rather less than that of tissue substance X and was about equal to that of the GSH–NEM reaction product Z′. The fast spot disappeared when NEM was added to the GSH stock solution but the mobility of the slow spot was unaffected. Thus the fast material probably contains a free thiol group whereas the slow moving spot evidently contains no such group.

It seemed of interest to investigate oxidation products of GSH arising in other experimental situations and to this end electrophoretic studies were made of ninhydrin-positive products resulting from (i) irradiation of a solution of GSH in phosphate buffer (pH 7·2) with ultraviolet light and from (ii) a reaction between GSH and napthalene-2-sulphonyl chloride (N–2–$SO_2Cl$).

(i) Woodward (1933) found that GSH was oxidised mainly to GSSG by ultraviolet light at pH 7 but some degradation of GSH also occurred. This reaction was reinvestigated with a solution of GSH (10 mg.) in pH 7·2 phosphate buffer (4 ml.) which was exposed in a 1 cm. quartz cell for 4 hours at 20° C. to unshielded light from the mercury arc of an Hanovia lamp, the mercury arc being about 6 inches from the cell. A suitable dilution of the irradiated solution (which had a strong smell of $H_2S$) was prepared with pH 7·2 buffer and aliquots with and without NEM were submitted to paper electrophoresis. Fig. 6A shows a typical separation of ninhydrin-positive materials resulting from this reaction (GSH + UV). For reference, GSH and its NEM reaction product have been included in the electropherogram. The main spot marked " GSH " seems to consist entirely of GSSG since its intensity was unchanged in a parallel run including NEM (not shown in Fig. 6) and no new spot of lower mobility appeared. A second prominent spot " X " having a lower mobility than the " GSH " spot occurred in the region generally occupied by tissue X spot. Its intensity and position were unaltered by NEM. Chromatographic analyses were made of unhydrolysed and hydrolysed " GSH " and " X " materials. Spot " GSH " contained only glutamic acid, glycine and cysteic acid and the chromatogram of the hydrolysate was identical with that shown in Fig. 2C for authentic GSH. A control analysis of authentic GSSG gave the same results. However it is clear from Fig. 6B and 6C that the substance " X " does not correspond to tissue substance X. It gave a single strong purple spot with an $R_f$ value of 0·37 in phenol (Fig. 6B). On hydrolysis, 3 spots were obtained corresponding to glutamic acid, glycine and $\alpha$-alanine with Rf values in phenol of 0·28, 0·39 and 0·60 respectively (Fig. 6C). Thus " X " could be identical with the tripeptide $\gamma$-glutamylalanylglycine which has been detected by Waley (1957) in bovine lens. DL–$\gamma$–glutamyl–DL–alanylglycine was synthesised according to Kermack and Matheson (1957) and subjected to paper electrophoresis, elution and chromatographic analysis as already described. It had the same electrophoretic mobility as peptide " X ". The unhydrolysed material had Rf 0·39 in phenol, and, on hydrolysis the peptide gave 3 spots due to glutamic acid, glycine and $\alpha$-alanine with Rf values of 0·30, 0·42 and 0·62 respectively in phenol and the spots had the same relative intensities as the spots seen in Fig. 6C. It is concluded that peptide " X " is $\gamma$-glutamyl-alanylglycine. It probably arose by loss of $H_2S$ from the cysteinyl residue of GSH with subsequent reduction of the resulting dehydroalanyl tripeptide to the alanyl derivative.

(ii) Saunders (1933) investigated a peculiar reaction which occurred between GSH and naphthalene-2-sulphonylchloride (N–$SO_2Cl$ or NSC). The latter failed to react in the ordinary way with the amino group of GSH. Instead it promoted an oxidation reaction and was itself reduced to naphthalene-2-sulphinic acid (N–2–$SO_2H$ or NSH). On stoichiometric grounds Saunders concluded that the main reaction product ought to be $GSO_2SG$ but his analysis of the ill-defined product failed to support this view.

This reaction was reinvestigated and after removal of NSH by precipitation with HCl, deep freezing and centrifugation, a portion of the yellowish acidic supernatant smelling strongly of $H_2S$ was diluted with phosphate buffer (pH 7·2) and examined electrophoretically. A typical separation is shown in Fig. 6A (GSH + NSC). Analysis of the main spot " GSH " revealed that it probably consists mainly of GSSG. Addition of NEM to the phosphate buffer solution resulted in the development of a Z′ spot, showing that some free thiol compound, probably GSH, is still present in the reaction mixture. As in the case of GSH + UV there appeared a very faint red spot just on the cathode side of the origin in the position to which authentic glycine migrates. These glycine spots cannot be seen clearly in the autopositive reproduction, Fig. 6A.

The nature of the "X" spot observed in this separation is of considerable interest although it is evidently quite different from tissue X spot. The unhydrolysed substance (Fig. 6D) gave a purple ninhydrin-positive spot with Rf0·32 in phenol. After acid hydrolysis, 2 spots were found in the chromatograms with Rf values in phenol of 0·28 and 0·39 due to glutamic acid and glycine respectively. Thus " X " is evidently a peptide consisting of glutamic acid and glycine. It could be either $\gamma$-glutamylglycine ($\gamma$-glugly) or $\gamma$-glutamylglycylglycine ($\gamma$-gluglygly). It is unlikely to be glycylglutamic acid since this substance would be expected to give a yellow colour with ninhydrin as do the glycyl dipeptides of glycine, serine and valine (Bowden, 1959). Samples of $\gamma$-glugly and $\gamma$-gluglygly were prepared by methods of King and Kidd (1949) and Kermack and Matheson (1957). These substances gave strong purple ninhydrin-positive spots with Rf values of 0·32 (glugly) and 0·29 (gluglygly) in phenol. After acid hydrolysis, $\gamma$-gluglygly gave two spots with Rf values in phenol of 0·39 and 0·27 due to glycine and glutamlc acid respectively. On paper electrophoresis, $\gamma$-glugly moved with the same mobility as authentic GSH whereas $\gamma$-gluglygly had the same mobility as the " X " product of Saunder's reaction. Thus $\gamma$-glutamylglycylglycine seems to be the main constituent of " X ". Its mode of formation is obscure. It may have arisen from GSH by the following reaction sequence :

It seems less probable that $\gamma$-gluglygly could have been formed in a transpeptidation reaction. In biochemical systems, $\gamma$-glutamylcysteine can transfer its glutamyl group to other amino acids present in the medium. For instance, Talalay (1954) observed the formation of $\gamma$-glutamylglycine and $\gamma$-glutamylglutamic acid in a solution of GSH (pH 7·4) which had been incubated for 1 hr at 37° C. with *Proteus vulgaris* or with cell-free extracts of this organism. Since traces of glycine have been detected among the products of reaction of GSH with NSC, $\gamma$-glutamylcysteine may be presumed to have been present at some stage in the reaction. Further work will be required to determine the precise mode of formation of $\gamma$-gluglygly in the NSC *in vitro* system.

Thus we have obtained no definite evidence for the occurrence *in vitro* of an oxidation product of GSH resembling tissue substance X. It will be noted however that traces of materials behaving like tissue substance X or GSH can be seen in Fig. 6, B, C, D and E. These traces may have arisen in the *in vitro* reactions described above but it seems more likely that they are due to small amounts of GSH which have been carried over from the main " GSH " spot in the paper sections containing " X " spot as selected for chromatographic analysis.

(f) *Stability of thiol peptide X in stored deep frozen phosphate extracts and tissues.*—Although a ninhydrin positive X spot can still be detected in electropherograms of aged extracts of hepatocarcinogen-treated rat livers and tumours, the mobility of the substance is no longer affected by NEM. An example of this can be seen in Fig. 7A. Apparently the thiol group has become masked probably through oxidation to disulphide or mixed disulphide. In view of the similar mobilities of GSH and GSSG under our conditions it was reasonable to conclude that XSH and XSSX would also have about the same mobilities.

An acid hydrolysate of X from a 2-month old extract of tannic acid-treated rat liver showed definite traces of aspartic acid (turquoise colour with ninhydrin), alanine and glycine as well as prominent spots due to glutamic and cysteic acids. A similar picture was given by an hydrolysate of X obtained from a benzopyrene-induced, tumour, with the exception that a spot corresponding to serine rather than to alanine appeared in the chromatograms.

Wieland (1954) has noted that alanine may arise in hydrolysates of GSSG (in the present work, however, alanine was never observed in hydrolysates of authentic GSSG) as well as the expected spots due to glycine, glutamic acid and cysteine. The occurrence of aspartic acid and serine in some of the aged X products suggests the formation of mixed disulphides of XSH with thiol peptides containing aspartic acid or serine. No evidence has been obtained for the presence of such peptides in any of our extracts nor have we observed the formation of any ninhydrin-positive products with themobility of X in stored deep frozen extracts of normal livers which were free of X when fresh.

Most of the stored extracts had been thawed, exposed briefly to the air and refrozen several times, factors which may have contributed to changes in X. The question as to whether X is a metabolically active material related to GSH seems to deserve further study, but it must not be forgotten that the presence of aspartic acid etc. might be explained on the basis of the *in vitro* formation of ninhydrin-negative substances such as acetyl-aspartic acid having the same mobility as X.

X, or a substance of similar mobility, has been found to survive for at least several months in deep frozen livers of hepatocarcinogen-treated rats. On the other hand, a specimen of normal guinea-pig liver which initially gave a strong X spot lost this material after several weeks at $-15°$ C. It is of interest that substance X from a normal guinea-pig liver extract which had been stored for 10 days at $-15°$ C. still responded completely to the addition of NEM. However, the intensity of the PE + GSH spot was unaffected by the NEM treatment. Evidently all the GSH had been converted to GSSG whereas the thiol group of substance X remained free.

2. *Nature of Y spot*

This small spot with mobility greater than that of aspartic acid was sometimes found in hepatocarcinogen-treated rat liver extracts and invariably in rat tumour extracts. It has not been extensively studied. It disappeared from electropherograms of an Rd/3 tumour phosphate extract to which NEM had been added, and the same effect was found with Y spot from normal guinea-pig liver extract. It has already been noted that a small spot with mobility was detected in electropherograms of an old specimen of GSH and similar products were observed in GSH solutions subjected to ultraviolet irradiation or to the action of naphthalene-2-sulphonyl chloride. These spots had about the same mobility as Y and disappeared when NEM was added.

A brownish spot appeared in the region of Y after spraying the electropherogram from tannic acid-treated rat liver phosphate buffer extract with aniline hydrogen phthalate spray (Partridge, 1949). This result suggests that a sugar-containing residue may be present in close association with Y.

3. *Nature of Z spot in various rat tumours, in the livers of tumour-bearing rats and in normal guinea-pig liver*

Extracts of all rat tumours investigated have been found to contain a ninhydrin-positive spot Z, examples of which are shown in Fig. 4 and in Fig. 7A.

Products of hydrolysis of Z spot from the BP tumour extract shown in Fig. 7A were chromatographed. The hydrolysate (Fig. 7B) contained appreciable amounts of aspartic, glutamic and cysteic acids and lesser amounts of ninhydrin-positive spots which have been tentatively identified as serine, glycine, proline, $\alpha$-alanine, valine, leucine, lysine and possibly arginine and histidine (compare reference chromatogram Fig. 7C). Practically the same amino acid picture was given by substance Z isolated from electropherograms of extracts of a rapidly growing Rd/3 tumour or from a slow-growing DMBA tumour.

Unhydrolysed extracts of Z from the three types of rat tumour were submitted to chromatography. In each case no spots due to free amino acids were detected All that could be seen was a very minute intensely purple spot running at the phenol front and having zero Rf in the butanol direction. Thus Z is evidently a protein or polypeptide-like material composed of at least 10 to 12 amino acids.

As already mentioned spots corresponding to Z were found in the livers of rats bearing transplantable tumours, in normal guinea-pig liver and also in normal rat kidney. However, when these spots were eluted and chromatographed there always appeared a single strong reddish ninhydrin-positive spot with Rf values similar to those of cysteine, as well as a minute purple spot running at the phenol front. When liver or kidney Z spots were hydrolysed and chromatographed an amino acid picture qualitatively rather similar to that given by tumour Z spot was obtained. The new spots apparently arose from the polypeptide moving at the phenol front. It was noted that the cysteic acid spot in hydrolysates of these Z spots was much more intense than in hydrolysates of material taken from a region lying on the origin side of Z spot. Unhydrolysed material from this position contained a polypeptide spot but no cysteine.

In spite of the rather sharp separation indicated in Fig. 7A it has been difficult to determine to what extent tumour Z spot has been contaminated with ninhydrin-positive material tailing from the origin towards Z spot. However it has been found possible to remove Z spot as well as all other amino acids spots by washing the electropherograms with several changes of deionised water. There remains a tail of material directed towards the anode from the origin which is revealed by staining with mercuric chloride-bromophenol blue or with ninhydrin. It probably consists of protein.

The compact and isolated nature of Z spot as seen in Fig. 7A suggests that Z spot is in fact a definite polypeptide entity unassociated with free amino acids. It is certain that tumour Z spot cannot be due merely to the presence of a free amino acid such as cysteine on a background of polypeptide or protein as seems to be the case with the Z spot material from other tissues.

### DISCUSSION

A thiol peptide X has been detected in the livers of rats injected with hepatocarcinogens at times when the free phosphoethanolamine content of the liver is maximal. Normal guinea-pig liver in contrast to normal rat liver already contains appreciable amounts of a substance similar to and apparently identical with X. Thiol peptide X contains the same amino acids as GSH but differs from GSH in having a lower electrophoretic mobility. X might be an isomer of GSH, a homologue of GSH, a higher peptide with the same amino acid constituents as GSH, or a derivative of GSH with some labile group. Various possibilities, some of which are being studied in detail, are outlined below.

With regard to possible isomers of GSH in which the mode of linkage of the constituent amino acids has been altered, little seems to be known. Du Vigneaud, Loring and Miller (1937) synthesised $\alpha$-glutamylycysteinyl-glycine. However this product (not known to occur naturally) retained its identity after liberation from its insoluble cuprous mercaptide complex. In taking account of optical isomers of GSH which might possibly have electrophoretic properties different from that of naturally occurring GSH, L-$\gamma$-glutamyl-L-cysteinylglycine, it is noted that the isomer D-$\gamma$-glutamyl-L-cysteinylglycine has been synthesised by Kögl and Akkerman (1946). The authors could find no polarimetric evidence for the occurrence of this substance in liver or tumour tissue. The fact that the D-isomer could be isolated unchanged from its insoluble cuprous mercaptide makes it unlikely that tissue substance X could be identical with this substance.

Ball, Revel and Cooper (1956) considered that $\gamma$-glutamylglutathione might be an intermediate in some transpeptidation reactions of GSH but apparently this peptide has not been isolated from biochemical systems nor has it been synthesised. It might fulfil the requirements for tissue substance X and indeed, after making allowance for the barbitone hydrolysis artifact, it would appear that the glutamic acid spot is more intense than either the glycine or cysteic acid spot in hydrolysates of X as compared with hydrolysates of authentic GSH (Fig. 2). In preliminary studies we have been able to effect separation of peptide X from GSH in the volatile component (pyridine-acetic acid-water) electrolyte recommended by Jirgl (1959). It is hoped with this method to obtain more exact information about the proportions of the constituent amino acids of X without interference from buffer artifacts.

The preponderance of glutamic acid in X might be due to the presence in the spot of a ninhydrin-negative glutamic acid derivative having the same mobility as X. One such contaminant might be pteroylglutamic (folic) acid which, on electrophoresis, separates into two fluorescent components which travel towards the anode. One of these components had the same mobility as X. It is quite possible that such a contaminant of X would only reveal itself by its contribution of extra glutamic acid in chromatograms of X hydrolysate.

Although a homologue of GSH ($GCH_2SH$) containing homocysteine in place of cysteine has been synthesised (Herrick and Todd, 1955 ; Gawron and Draus, 1959) this substance has apparently not been found in biochemical systems. Such a compound with an extra $-CH_2-$ group in the thiol side chain might well have properties similar to X. It might not be possible to distinguish $GCH_2SH$ from GSH by chromatography of its hydrolysis products since homocysteic acid has about the same Rf values as cysteic acid in Bowden's system. Like GSH, the homologue would be expected to form an insoluble cuprous mercaptide. However, Herrick and Todd (1955) found that the cuprous mercaptide of L-$\gamma$-glutamyl-DL-homocysteinylglycine is in fact easily soluble in water so the possibility cannot be ruled out that thiol peptide X, if not $GCH_2SH$, may at least be capable of forming a water-soluble cuprous derivative and so be lost in the cuprous mercaptide precipitation procedure.

There has been speculation as to the existence of a variety of interrelated GSH derivatives including compounds with pyrrolidine and thiazoline rings (Calvin, 1954 ; Isherwood, 1959). Pyrrolidine-imonium (PI) or hydroxypyrrolidine (HP) forms of GSH proposed by Calvin (1954) have free thiol groups but the $\alpha$-amino group of the glutamyl residue has reacted with the $\gamma$-carbonyl group with loss of water (PI) or by simple addition (HP). According to Calvin, HP could readily revert to the normal open chain form of GSH in the presence of hydrogen ions. Thus the reaction of tissue substance X with NEM, its colour reaction with ninhydrin-acetic acid and the apparent disappearance of X in the acidic conditions required for cuprous mercaptide formation could be accounted for if X possessed the hydroxypyrrolidine structure.

Thiol peptide X might be a GSH derivative with a substituent not readily detectable chromatographically or easily lost during handling. Dubin and Rosenthal (1960) reported the isolation of a conjugate of GSH with spermidine from *E. coli*. From the available information it would appear that this complex consists of a molecule of GSSG which has reacted through its COOH groups with the amino groups of spermidine. The conjugate ran as a single spot but after hydrolysis it gave four spots due to glycine, glutamic acid, cysteic acid and spermidine. In view of the elevated levels of PE which occur in hepatocarcinogen-treated rat liver it seemed that X might be a similar conjugate of GSH with PE or ethanolamine. No chromatographic evidence has yet been obtained in favour of the existence of such a conjugate, but it is highly likely that PE or ethanolamine residues would fail to survive the hydrolytic and freeze-drying procedures employed in the present study.

There are some indications in the literature that substances like thiol peptide X have been detected in other situations. Thus Spicer and Weise (1955) reported that a cationic fraction of guinea-pig or rabbit liver, submitted to paper electrophoresis at pH 4·6, gave two unidentified ninhydrin-positive spots which stood in the same relation to GSH, glutamic and aspartic acids as did substances X and Y of the present study.

Waley (1956) found that an acidic fraction of calf lens contained several peptides consisting of glutamic acid, cystine (or cysteine) and glycine. These materials were not identical with GSH and they appeared to contain differing molar ratios of glycine to glutamic acid. Waley considered that one of these peptides might carry a phosphate group.

Keir and Davidson (1956) reported that the cytidine monophosphate fraction of rabbit tissues invariably gave a reaction with ninhydrin. When the fraction was submitted to electrophoresis the ninhydrin-positive constituent moved ahead of cytidine monophosphate. On hydrolysis, the unknown constituent yielded glutamic acid, cystine and glycine together with an unidentified base which absorbed ultraviolet light.

Whatever the nature of X and Y may be, it appears that X at least may result from some disruption of the normal course of GSH metabolism in rat liver through the influence of hepatocarcinogens or by the presence of a remote tumour. Substances X and Y may themselves play some part in tumour metabolism. X may be a normal trace derivative of GSH which appears in increased amount in rat liver under the stress conditions imposed by hepatocarcinogens or tumours. Schacter and Law (1956) found that there is an increased requirement for GSH from the liver, accompanied by an increased output of GSH by the liver, during the growth of an ascitic form of lymphocytic leukaemia in mice.

It is of considerable interest that a substance similar to thiol peptide X is already present in appreciable amounts in the liver of normal guinea-pig, a species which is refractory to hepatocarcinogenesis. Moreover, chromatographic evidence has been obtained that 3'MeDAB injection failed to increase the level of free PE in guinea-pig liver (Neish, unpublished results) whereas this hepatocarcinogen induced a marked increase of PE in rat liver. These pronounced differences in behaviour of X and PE in the livers of rat and guinea-pig may well be connected with the susceptibility of the former and the refractoriness of the latter species to hepatocarcinogenesis.

A significant relationship seems possible between the high level of GSH in hepatocarcinogen-treated rat liver (at least in the initial stages ; Fiala, 1958) and the elevated PE content. Henneman, Altschule and Goucz (1955) found a decrease in the level of inorganic phosphate in the serum after injection of GSH into humans which suggests that the high level of GSH may be promoting phosphorylation reactions. Conversely, according to Jocelyn (1959) low GSH levels may depress phosphorylation. Some evidence for this is found in the work of Ling and Chow (1954) who showed that the blood and tissue levels of phospholipids are abnormally low in Vitamin $B_{12}$-deficient (low GSH) rats. It is interesting that the enzyme alkaline phosphatase which catalyses the hydrolysis of PE (PE may be the natural substrate for this enzyme, according to Dent, 1956) is said to activated by GSSG and inhibited by GSH (Fell and Danielli, 1944).

Thus the increased level of GSH in the liver of rats injected with 3′-MeDAB might be expected to favour the accumulation of PE which has in fact been observed (Neish and Rylett, 1960).

Vollmer (1954) stated that GSH is lost from red blood cells in the presence of methaemoglobinogenic agents. Since it is known that 3′-MeDAB induces methaemoglobinemia in the rat (Neish, 1956) it seemed possible that the formation of thiol peptide X in rat liver might be connected with some disturbance in liver GSH metabolism associated with the methaemoglobinogenic action of the azo dye. However, such an effect can probably be ruled out because the equally potent hepatocarcinogen, 4′-EtDAB, behaves exactly like 3′-MeDAB with regard to the accumulation of PE and the formation of thiol peptide X in rat liver although, compared with 3′-MeDAB on a mole for mole basis, it has no methaemoglobinogenic activity in the rat (Neish, 1959).

The substance Z which has been found in extracts of rapid and slow growing transplantable rat tumours and of benzopyrene-induced rat tumours seems to be a protein or polypeptide. Borsook *et al.* (1949) obtained a polypeptide consisting of 15 amino acids from the livers of various species. Several acidic peptide-nucleotide complexes have been encountered by Steinberg *et al.* (1960) in mammalian liver and Ågren (1960) has obtained an acid-soluble nucleotide-linked peptide from trichloracetic acid extracts of ascites tumour cells. On hydrolysis, this peptide gave particularly strong gly, glu, ser and asp spots, weaker spots due to leu, iso-leu, lys, arg, threo and at least 4 more spots which were not identified.

Tumour substance Z may be compared with the toxohormone of Nakahara and Fukuoka (1958). Toxohormone is said to be produced by all malignant tumours and its major amino acid components are stated to be ala, pro, asp, arg, Øala, lys, leu and glu. A toxohormone-like material isolated from the urine of cancer patients by Fuchigami, Umeda and Ono (1956) contained asp, glu, ser, threo, pro, ala, lys, val, leu, Øala, cystine and gly all of which with the exception of threo and Øala appear to occur in Z. Whether Z further resembles toxohormone in having catalase-inhibiting properties remains to be determined.

It is of interest that hepatocarcinogens induce the formation of a thiol peptide X and of excessive amounts of phosphoethanolamine in rat liver. Rat sarcomas appear to have a higher level of PE than normal rat muscle tissue (Neish, unpublished results) and they also contain thiol peptide X which is not present in normal muscle. These observations suggest that it might be worthwhile to examine the possibility of the emergence of tumour-type metabolic patterns even in the early stages of the action of a carcinogen on its target organ. Recently, Kögl *et al.* (1960) stated that the lecithin fraction of primary rat tumours induced by 4-dimethylaminoazobenzene incorporated considerably more $^{32}P$ *in vitro* than did the phosphatidylethanolamine fraction. The opposite situation was found with normal liver tissue. Thus a shift in the metabolic activities of both phosphatides appears to occur during development of liver tumours. It has already been noted (Neish and Rylett, 1960) that a possible explanation for the accumulation of PE in the livers of hepatocarcinogen-injected rats lies in a carcinogen-induced failure of the enzymic mechanism for phosphatidylethanolamine synthesis. Kögl's work suggests that a similar defect may be present in the azo-dye induced tumours. Thus an early effect of the carcinogen may persist through the course of hepatocarcinogenesis.

### SUMMARY

1. The livers of hepatocarcinogen-treated rats and of normal guinea-pigs contain a substance X which is apparently a thiol peptide closely related to glutathione. A similar substance has been detected in transplantable and induced rat tumours together with a substance Y which may also be a thiol peptide. Attempts to characterise X fully have not yet been successful but some pointers towards its possible nature have been obtained.

2. All the rat tumours investigated contained a substance Z which seems to be a protein or polypeptide containing at least 10 to 12 amino acids.

Thanks are due to Miss P. Ingleton for a gift of the benzopyrene-induced rat tumours and for the guinea-pig material used in this study and to Dr. J. F. Barker for supplying DMBA tumour material. We should like to thank Professor H. N. Green for his encouragement and interest in this work.

### REFERENCES

ÅGREN, G.—(1960) *Acta chem. scand.*, **14**, 2065.
BALL, E. G., REVEL, J. P. AND COOPER, O.—(1956) *J. biol. Chem.*, **221**, 895.
BENESCH, R., BENESCH, R. E., GUTCHO, M. AND LAUFER, L.—(1960) *Science*, **123**, 981.
BLASS, J., LE COMTE, O. AND POLONOVSKI, J.—(1954) *Bull. Soc. Chim. biol., Paris*, **36**, 627.
BORSOOK, H., DEASY, C. L., HAAGEN-SMIT, A. J., KEIGHLEY, G. AND LOWY, P. H.—(1949) *J. biol. Chem.*, **179**, 705.
BOWDEN, C. H.—(1959) *Clin. Chim. Acta*, **4**, 539.
BOYLAND, E. AND MAWSON, E. H.—(1938) *Biochem. J.*, **32**, 1460.
CALVIN, M.—(1954) 'Glutathione'. Ed. S. Colowick *et al.* New York (Academic Press Inc.), p. 3.
DENT, C. E.—(1956) Ciba Symposium—Bone Structure and Metabolism. London (Churchill), p. 266.
DUBIN, D. T. AND ROSENTHAL, S. M.—(1960) *Fed. Proc.*, **19**, Part 1, p. 1.
DU VIGNEAUD, V., LORING, H. S. AND MILLER, G. L.—(1937) *J. biol. Chem.*, **118**, 391.
FELL, H. B. AND DANIELLI, J. F.—(1944) *Bull. War Med.*, **4**, 253.
FIALA, S.—(1958) *Nature, Lond.*, **182**, 257.
FRIEDMANN, E., MARRIAN, D. H. AND SIMON-REUSS, I.—(1949) *Brit. J. Pharmacol.*, **4**, 105.
FUCHIGAMI, A., UMEDA, M. AND ONO, T.—(1956) *Gann.*, **47**, 295.
GAWRON, O. AND DRAUS, F.—(1959) *J. org. Chem.*, **24**, 1392.
GREGORY, J. D.—(1955) *J. Amer. chem. Soc.*, **77**, 3922.
GUTCHO, M. AND LAUFER, L.—(1954) 'Glutathione'. Ed. S. Colowick *et al.* New York (Academic Press Inc.), p. 79.
HENNEMAN, D. H., ALTSCHULE, M. D. AND GOUCZ, R. M.—(1955) *Metabolism*, **4**, 433.
HERRICK, E. C. AND TODD, C. W.—(1955) *U.S. Pat. Syst. Leafl.*, No. 2,723,972 and 2,723,973.
ISHERWOOD, F. A.—(1959) *Biochem. Soc. Symp.* No. 17, Glutathione. London (Cambridge University Press), p. 13.
JIRGL, V.—(1959) *Experientia*, **15**, 235.
JOCELYN, P. C.—(1959) *Biochem. Soc. Symp.* No. 17, Glutathione. London (Cambridge University Press), p. 60.
KEIR, H. M. AND DAVIDSON, J. N.—(1956) *Biochem. J.*, **63**, 23P.
KERMACK, W. O. AND MATHESON, N. A.—(1957) *Ibid.*, **65**, 45.
KING, F. E. AND KIDD, D. A. A.—(1949) *J. chem. Soc.*, p. 3315.

Kögl, F. and Akkerman, A. M.—(1946) *Rec. Trav. chim. Pays-bas*, **65**, 216, 225.
*Idem*, Smak, C., Veerkamp, J. H. and Van Deenen, L. L. M.—(1960) *Z. Krebsforsch.*, **63**, 558.
Ling, C. T. and Chow, B. F.—(1954) *J. biol. Chem.*, **206**, 797.
Nakahara, W. and Fukuoka, F.—(1958) *Advanc. Cancer Res.*, **5**, 157.
Neish, W. J. P.—(1956) *Nature, Lond.*, **178**, 1350.—(1959) *Naturwissenschaften*, **46**, 535.
*Idem* and Rylett, A.—(1960) *Brit. J. Cancer*, **14**, 737.
Partridge, S. M.—(1949) *Nature, Lond.*, **164**, 443.
Sauberlich, H. E. and Baumann, C. A.—(1951) *Cancer Res.*, **11**, 67.
Saunders, B. C.—(1933) *Biochem. J.*, **27**, 397.—(1934) *Ibid.*, **28**, 1977.
Schacter, B. and Law, L. W.—(1956) *J. nat. Cancer Inst.*, **17**, 391.
Spicer, S. S. and Weise, V.—(1955) *Enzymologia*, **17**, 263.
Steinberg, D., Vaughan, M., Sherman, F. G. and O'Dell, B. L.—(1960) *Biochim. biophys. Acta*, **40**, 225.
Talalay, P. S.—(1954) *Nature, Lond.*, **174**, 516.
Vollmer, E. P.—(1954) 'Glutathione'. Ed. S. Colowick *et al.* New York (Academic Press Inc.), p. 186.
Waelsch, H. and Rittenberg, D.—(1941) *J. biol. Chem.*, **139**, 761.
Waley, S. G.—(1956) *Biochem. J.*, **64**, 715.—(1957) *Ibid.*, **67**, 172.
Wieland, T.—(1954) 'Glutathione'. Ed. S. Colowick *et al.* New York (Academic Press Inc.), p. 55.
Woodward, G. E.—(1933) *Biochem. J.*, **27**, 1411.